# To multicellularity and back again: Description of two new coccoid genera (*Portococcus* gen. nov. and *Pseudanabaenococcus* gen. nov.) in the basal “filamentous” order Pseudanabaenales, Cyanobacteria

**DOI:** 10.1111/jpy.70130

**Published:** 2026-02-17

**Authors:** Otakar Strunecký, Eliška Kozlíková‐Zapomělová, Jitka Jezberová, João Morais, María Alicia Toledo Lemus, Lenka Štenclová, Jeffrey R. Johansen, Kateřina Čapková, Vitor M. O. Vasconcelos, Jan Mareš

**Affiliations:** ^1^ Faculty of Fisheries and Protection of Waters, South Bohemian Research Center of Aquaculture and Biodiversity of Hydrocenoses, Institute of Aquaculture and Protection of Waters, University of South Bohemia in České Budějovice České Budějovice Czech Republic; ^2^ Biology Centre of the Czech Academy of Sciences Institute of Hydrobiology České Budějovice Czech Republic; ^3^ CIIMAR/CIMAR, Interdisciplinary Centre of Marine and Environmental Research University of Porto Porto Portugal; ^4^ Department of Botany, Faculty of Science University of South Bohemia České Budějovice Czech Republic; ^5^ Department of Pharmaceutical Biology, Institute of Pharmacy Freie Universität Berlin Berlin Germany; ^6^ Department of Biology John Carroll University University Heights Ohio USA

**Keywords:** cyanobacteria, freshwater, polyphasic taxonomy, *Portococcus*, Pseudanabaenales, *Pseudanabaenococcus*, terrestrial, unicellular

## Abstract

Despite recent efforts in taxonomic revision of phylogenetically basal photosynthetic cyanobacteria, cryptic diversity and recurrence of simple plesiomorphic morphotypes has continued to appear in phylogenies with poorly characterized “*Synechococcus*” and “*Pseudanabaena*” strains. Herein, one of the prominent undefined unicellular lineages was resolved as a monophyletic group of taxa that have lost multicellularity within the otherwise filamentous order Pseudanabaenales. Genome sequencing coupled with the classical polyphasic taxonomic analysis based on the 16S rRNA gene and the ITS rRNA region sequence comparisons, light and transmission electron microscopy, and source habitat record have congruently supported the description of two novel genera, *Portococcus*, with four new species, and *Pseudanabaenococcus*, with a single new species. The whole‐genome phylogeny was essential for the accurate assessment of phylogenetic relationships between the genera and families and is hereby highly recommended as a new standard integrated in the polyphasic taxonomy of cyanobacteria. Records of the new taxa from a variety of freshwater habitats and one terrestrial cave habitat in geographically distant regions have indicated the need for further investigation to discover the full extent of cryptic diversity in these deep‐branching clades. The loss of filamentous forms within the first group in cyanobacterial evolution that had invented it offers an excellent model for future study of the genetic and physiological mechanisms of early prokaryotic multicellularity.

**Abbreviations**
AAI: average amino acid identity
AIC: Akaike information criterion
ANI: average nucleotide identity
BI: Bayesian inference
CAS: Czech Academy of Sciences
CIIMAR: Centro Interdisciplinar de Investigação Marinha e Ambiental
dDDH: digital DNA:DNA hybridization
DIC: differential interference contrast
ESS: estimated sample size
GTDB: Genome Taxonomy Database
GTR + I + G: general time reversible model with a proportion of invariable sites estimation, and gamma distribution
ICN: International Code of Nomenclature for algae, fungi, and plants
ICNP: International Code of Nomenclature of Prokaryotes
ITS: internal transcribed spacer
LEGE‐CC: Blue Biotechnology and Ecotoxicology Culture Collection
LM: light microscopy
MCMCMC: Metropolis‐coupled Markov chain Monte Carlo
ML: maximum likelihood
NCBI: National Center for Biotechnology Information
PCC: Pasteur Culture Collection
PCR: polymerase chain reaction
PROTGAMMAILG: an improved general amino acid replacement matrix with a proportion of invariable sites estimation, and gamma distribution
PSRF: potential scale‐reduction factor
SAG: Culture Collection of Algae (Göttingen University)
TEM: transmission electron microscopy

## INTRODUCTION

Advancements in molecular biology and whole genome sequencing have significantly enhanced our understanding of cyanobacterial phylogenetic taxonomy, leading to the recent redefinition of deep‐branching lineages (Strunecký et al., 2023). One of these lineages is the order Pseudanabaenales, recently split into two distinct families: Pseudanabaenaceae and Thalassoporaceae (Strunecký et al., 2023). The new family Thalassoporaceae has been closely linked to marine and brackish environments and was clearly separated by its phylogenetic position. Although Pseudanabaenales as a whole exhibit a wide ecological distribution—from freshwater habitats (Willame et al., 2006) through brackish water (Acinas et al., 2009) to saltwater environments (Konstantinou et al., 2021)—and span tropical (Khan et al., 2018) to polar regions (Khan et al., 2017), their morphological diversity has been considered relatively limited. This perceived limitation was further enhanced by a major taxonomic overhaul of the order Pseudanabaenales, driven primarily by multilocus phylogenetic analyses, which led to the reclassification of several families (e.g., Spirulinaceae promoted to Spirulinales; Komárek et al., 2014) and proposals for their narrower definition (Mai et al., 2018). Consequently, a notable reduction in the number of genera within Pseudanabaenales has occurred in recent years (Strunecký et al., 2023).

The majority of sequenced species described within the family Pseudanabaenaceae have been clustered into a single clade, forming a relatively homogeneous group (Aleksovski et al., 2024). A key distinguishing feature of Pseudanabaenaceae, particularly when compared to thin filamentous cyanobacteria such as in Leptolyngbyaceae, Oculatellaceae, and Nodosilineaceae, is the presence of chains of cylindrical cells visually separated by so‐called “hyaline bridges,” with prominent constrictions at cell walls that typically represent about one third to one fourth of the cell diameter in longitudinal cross‐sections. Despite the recent split, the morphological traits of the newly erected sister family Thalassoporaceae closely resemble those of Pseudanabaenaceae (Konstantinou et al., 2021). Pseudanabaenales are generally characterized by barrel‐shaped cells measuring 1–2 μm in width and 1–9 μm in length (Aleksovski et al., 2024). These motile cyanobacteria (Yamamoto et al., 2021) are further equipped with parietal thylakoids and gas vesicles (Mareš et al., 2019). The cell length frequently varies among individual filaments, exhibiting either an isodiametric form or, more commonly, a significant proportion of cells that are longer than wide. These cyanobacteria typically thrive in periphytic mats or planktonic communities within their aquatic habitats (Kling et al., 2012).

A particularly conspicuous feature of Pseudanabaenales is the highly variable number of cells within their trichomes. Many Pseudanabaenalean strains exhibit brevitrichomy (Pinevich et al., 1997), characterized by short trichomes with a relatively low number of cells, typically ranging from (4)6 to 15(25) cells (Aleksovski et al., 2024; Khan et al., 2018; Nishizawa et al., 2010; Yamamoto et al., 2021). However, these short‐filamented strains can occasionally form trichomes with significantly longer chains of cells during cultivation, suggesting that specific yet unidentified environmental factors may drive this phenotypic plasticity. The presence of short and easily fragmented trichomes may reflect a relatively primitive form of cell interconnection compared to derived filamentous groups such as Oscillatoriales, since Pseudanabaenales most likely represent the first instances of multicellularity in cyanobacteria (Schirrmeister et al., 2011). From the brevitrichomy point of view, it could also be argued that some of the previously described short‐filamented species attributed to *Borzia* or *Hormoscilla* might belong to Pseudanabaenaceae. However, the cell width of these species is typically more than three times larger than in the Pseudanabaenaceae (Bohunická et al., 2015; Komárek & Anagnostidis, 2005), making their future reclassification into the Pseudanabaenales improbable. In contrast, several strains originally isolated as *Synechococcus* spp. due to their unicellular (PCC 6802) or up‐to‐three‐cells‐long morphology (PCC 6901 and 6903) were later reassigned to the genus *Pseudanabaena* (Rippka et al., 1979). This reclassification was prompted by the appearance of gas vesicles and the presence of distinctive refractile “bipolar granules,” typical features of *Pseudanabaena* (Guglielmi & Cohen‐Bazire, 1984). However, the formation of short chains of connected cells following cell division is quite common even among other unicellular cyanobacteria (Komárek et al., 1999). This phenomenon has often led to chains of up to approximately eight cells that eventually disintegrate, a behavior observed both in natural environments and in controlled laboratory cultures, as described for former *Synechococcus* species (Komárek et al., 2020).

Frequent overlap between short chains and true filaments highlights the challenges in distinguishing between coccoid and filamentous forms in cyanobacteria. The boundaries between these morphological types are often blurred, reflecting the complexity and plasticity inherent in cyanobacterial biology. The transitional nature of these forms emphasizes the need for a deeper understanding of cellular and genetic factors that drive morphological variability in cyanobacteria. Such complexities not only complicate taxonomic classification but also suggest that morphological distinctions may not always correspond to distinct evolutionary lineages, highlighting the importance of integrative approaches that combine morphological, genetic, and ecological data for accurate identification and classification of cyanobacterial taxa. The whole problem has been further complicated by the low resolution of 16S rRNA gene phylogenies above the genus level (Mareš, 2018) as well as the still insufficient taxon sampling and whole‐genome data in many deep‐branching cyanobacterial groups of the former Synechococcales.

In this study, we have undertaken steps toward a deeper understanding of the evolutionary routes between uni‐ and multicellularity in Pseudanabaenales by identifying and taxonomically describing the unequivocally unicellular types. We have included newly acquired whole‐genome data that allow reliable reconstruction of the relationships among unicellular versus filamentous representatives within the order, paving the way for future functional studies in the early evolution of multicellularity.

## MATERIALS AND METHODS

### Sampling and strain isolation

Unialgal cyanobacterial strains were isolated from the natural samples by inoculating a small amount of the collected material to solidified Z medium (Zehnder, unpublished data as cited in Staub, 1961) or liquid WC medium (Guillard & Lorenzen, 1972). Subsequently, cells were transferred to fresh plates or diluted until clonal strains were obtained. A list of all strains analyzed is given in Table 1. The cyanobacteria were long‐term cultivated in liquid and agar‐solidified BG11 media (Rippka et al., 1979), liquid WC medium, or liquid Z medium (Zehnder in Staub, 1961) at 18°C and at a 16:8 h light:dark cycle. Using the methods described above, three new strains of unicellular Pseudanabaenaceae were successfully isolated and are being maintained in the culture collections of the Institute of Hydrobiology, Biology Centre of the Czech Academy of Sciences, and LEGE‐CC, Centro Interdisciplinar de Investigação Marinha e Ambiental (CIIMAR), University of Porto (Table 1). Additional strains of unicellular Pseudanabaenaceae were purchased from available public culture collections: Pasteur Culture Collection (PCC) and Culture Collection of Algae (SAG). For preparation of herbarium‐type specimens of the newly described taxa, biomass from the respective strains was harvested on GF/C filters (1.2 μm porosity, Whatman), gently air‐dried, and deposited in the CBFS Herbarium (Thiers 2019) at the University of South Bohemia, České Budějovice, Czech Republic under CBFS accession numbers A‐264–A‐268 (see descriptions of the new taxa).

**TABLE 1:** Reference strains and holotypes of new taxa analyzed in this study.

Species	Strain	Year of isolation	Habitat	Locality	Herbarium voucher
*Portococcus lusitanicus*	LEGE 16609	2020	Pool in a mountain stream	Barrancos dos Pisoes, Serra de Monchique, Portugal	CBFS A‐264
*Portococcus limneticus*	SE‐L21B6	2020	Freshwater post‐mining lake	Lake Senftenberg, Germany	CBFS A‐265
*Portococcus sanctae‐mariae*	UPSI	2023	Wet rock, limestone cave	Wetterloch Cave, Schafberg, near to St. Wolfgang, Austria	CBFS A‐268
*Portococcus stanieri*	PCC 6802	1968	Freshwater pond	California University, Berkeley, United States	CBFS A‐266
*Pseudanabaenococcus habilitatus*	SAG 2387	1973	Mountain stream	Harz Mountains, Westerhoefer Creek, Germany	CBFS A‐267

### Morphology and ultrastructure

Morphology of all strains was documented by light microscopy (LM) using an Olympus BX‐51 microscope equipped with Nomarski DIC optics (up to 1000× magnification), DP‐72 digital camera, and Olympus cellSens Standard v. 2.1 image analysis software for photographic documentation and measurements (>50 cells of each strain were measured). For transmission electron microscopy (TEM) studies, the biological material of cyanobacteria was fixed with 6% glutaraldehyde and kept at room temperature. Samples were washed with 0.05 M phosphate buffer (pH 7.2) and postfixed with 2% osmium tetroxide in the same buffer at room temperature for 2 h, and then repeatedly washed with 0.05 M phosphate buffer. Finally, cells were dehydrated with a graded isopropanol series and embedded in Spurr's resin (Spurr, 1969) using propylene oxide as an intermediate stage. Ultrathin sections were stained with uranyl acetate and lead citrate and observed in a Jeol JEM 1400 transmission electron microscope at 80 kV.

### DNA isolation and 16S rRNA gene sequencing

Previously isolated strains from cyanobacterial culture collections as well as newly isolated strains (Table 1) were utilized for DNA sequencing in this study. The two different laboratories working on this collaborative project used different DNA extraction and sequencing methods, and we have described both these sets of methods below.

Total genomic DNA of LEGE stains was extracted from a fresh pellet of 50 mL of culture using the commercial NZY Plant/Fungi gDNA Isolation kit (NZYTech, Lisboa, Portugal), according to the manufacturer's instructions. Prior to DNA extraction, cyanobacterial cells, once adequately dried, were placed in a chilled mortar, flash frozen using liquid nitrogen, and subjected to mechanical lysis using a pestle until cells were pulverized into a fine powder. The resulting powder was promptly scraped into a sterile 2‐mL Eppendorf tube for DNA extraction. Total genomic DNA from the remainder of our unialgal strains was isolated using DNeasy PowerSoil Pro Kit (Qiagen, Venlo, Netherlands) or NucleoSpin Soil Kit (Macherey‐Nagel, Düren, Germany) according to the manufacturer's instructions or amplified from single filaments using multiple‐displacement amplification with the Repli‐G Mini Kit (Qiagen, Hilden, Germany) as described previously (Mareš et al., 2014).

A section of the rRNA operon containing the partial 16S rRNA gene and the 16S–23S internal transcribed spacer (ITS) rRNA region was amplified using the primers 359F (5′‐GGGGAATTTTCCGCAATGGG‐3′; Nübel et al., 1997) and 23S30R (5′‐CTTCGCCTCTGTGTGCCTAGGT‐3′; Wilmotte et al., 1993). The polymerase chain reaction (PCR) mixture contained the template DNA, 10 pmol of each primer, and 50 μL of commercial PCR mix with Taq polymerase (Plain PPP Master Mix, Top Bio, Prague, Czech Republic). The PCR was conducted under the following conditions: an initial denaturation at 94°C for 10 min, followed by 40 cycles of 30 s at 94°C, 1 min at 56°C, and 3 min at 72°C, with a final extension at 72°C for 10 min, and cooling to 4°C. Successful PCR amplification was confirmed by electrophoresis of a subsample on a 1.5% agarose gel stained with Gel Red. All PCR products were purified using the QIAquick PCR Purification Kit (Qiagen) and sequenced using the primers 359F, 1492R (5′‐TAC GGY TAC CTT GTT ACG ACTT‐3′), 810R (5′‐GTT ATG GTC CAG CAA AGC GCC TTC GCCA‐3′), and 23S30R (Strunecký et al., 2014) at Eurofins Genomics (Ebersberg, Germany) or SeqMe (Dobříš, Czech Republic). The 16S rRNA gene and ITS rRNA region sequences were deposited to GenBank under accession numbers PV263562‐PV263567 and PV292134‐ PV292143, respectively.

### Genome sequencing and assembly

Quality of the gDNA of LEGE strains was evaluated in a DS‐11 FX Spectrophotometer (DeNovix, Wilmington, Delaware, United States) and 1% agarose gel electrophoresis, before genome sequencing. Genomes were sequenced at MicrobesNG using the Illumina platform with 2 × 250 bp paired‐end libraries. Because the cyanobacterial cultures were not axenic, the contigs obtained were analyzed using the binning tool MaxBin2 v. 2.2.4 (Wu et al., 2016) and CheckM v. 1.0.18 (Parks et al., 2015) within Kbase software (Arkin et al., 2018) to obtain only cyanobacterial contigs. MaxBin2 separates contigs into different bins by employing an Expectation–Maximization (EM) algorithm that integrates nucleotide composition, abundance information (inferred from read depth), and phylogenetic marker genes. The quality of the resulting genome bins was assessed using CheckM v. 1.0.18 (Parks et al., 2015). To ensure that downstream analyses were focused exclusively on cyanobacteria, only contigs classified as cyanobacterial were retained; all non‐cyanobacterial bacterial contigs were excluded.

Total genomic DNA isolated from the unialgal cultures of the remaining strains was quantified using a Qubit 4.0 (Thermo Fisher Scientific, Waltham, Massachusetts, United States) fluorimeter, and sent for commercial de novo genome sequencing (SeqMe, Dobříš, Czech Republic) using an Illumina MiSeq Pair‐End library with 250 bp reads, 350 bp average insert length, and 1.2 Gbp data yield. The raw data from Illumina were trimmed, assembled, binned, and taxonomically classified using the nf‐core/mag metagenomics pipeline (Krakau et al., 2022) installed on the server of the Institute of Hydrobiology, Biology Centre of the Czech Academy of Sciences (CAS). Genome sequence data associated with strains used in this study were deposited in National Center for Biotechnology Information (NCBI) GenBank under BioProject numbers: SAMN47198195–SAMN47198203 and SAMN48121352–SAMN48121354.

### Phylogenetic analysis

The sequences of the 16S rRNA gene were aligned using MAFFT (mafft.cbrc.jp; Katoh & Toh, 2010), and the alignments were visually inspected with BioEdit 7.0.1 (Hall, 1999). The final 16S rRNA gene alignment comprised 188 sequences with 1184 columns. The optimal nucleotide substitution model for phylogenetic analysis was determined using jModelTest 2 (Darriba et al., 2012) based on the Akaike information criterion (AIC) as the general time‐reversible model with invariant sites and gamma distribution (GTR + I + G). Phylogenetic reconstruction using this substitution model was conducted via Bayesian inference (BI) in MrBayes 3.2.6 (Ronquist et al., 2012). The BI analysis employed metropolis‐coupled Markov chain Monte Carlo (MCMCMC) with three heated chains and one cold chain across two independent runs, processed for 10 million generations. Tree sampling occurred every 1000 generations until the likelihood values stabilized, achieving an average standard deviation of split frequencies at 0.01. A burn‐in of the initial 25% of generations allowed stabilization of the likelihood values in the sampled trees, and a 50% majority‐rule consensus tree was constructed with estimated posterior probabilities for the branches. Maximum likelihood (ML) analysis using the GTR + I + G model was performed in RAxML v 8.0.0 (Stamatakis, 2014) with 1000 bootstrap replicates at the supercomputing facility of the Faculty of Science, University of South Bohemia. Bootstrap values from ML were mapped onto the BI tree.

The multilocus alignment for phylogenomic analysis was generated using the workflow provided by the Genome Taxonomy Database toolkit (GTDB‐Tk; Parks et al., 2020; Parks et al., 2018) and the GTDB‐Tk v. 2.4.0 (Chaumeil et al., 2022) release from April 24, 2024, which included 3519 quality‐checked cyanobacterial genomes. A representative selection of 756 genomes was chosen to reflect the cyanobacterial tree of life according to Strunecký et al. (2023). In total, 759 genomes from the GTDB and three new genomes of unicellular Pseudanabaenaceae sequenced in this study were used to create an alignment based on 120 concatenated conserved bacterial markers, resulting in a protein alignment that was 5035 amino acids long.

A phylogenomic tree was constructed from the alignment in RAxML using the PROTGAMMAILG model, with branch support estimated from 1000 nonparametric bootstrap replicates. Phylogenetic reconstruction using the same substitution model was also performed with BI in MrBayes 3.2.6 (Ronquist et al., 2012). The BI analysis employed MCMCMC with three heated chains and one cold chain across two independent runs, processed for 10 million generations. Trees were sampled every 1000 generations until likelihood values stabilized, achieving an average standard deviation of split frequencies at 0.06, with average PSRF and ESS values of 1.01 and 1520, respectively. A burn‐in of the initial 25% of generations allowed stabilization of the likelihood values within the sampled trees, and a 50% majority‐rule consensus tree was constructed with estimated posterior probabilities for the branches. Posterior probabilities from the BI analysis were then mapped onto the multilocus ML tree.

### ITS rRNA region analysis

The ITS rRNA regions of all five target strains (representing five different species of unicellular Pseudanabaenaceae) in this study were obtained, complete with the leader region of the ribosomal operon that binds with the D2 region of the 16S–23S ITS rRNA and the 23S–5S ITS rRNA region that binds with the D4 and D5 regions of the 16S–23S ITS rRNA region. With the use of these additional regions, it was possible to estimate with greater confidence the structures at the beginning and end of the 16S–23S ITS rRNA region. The conserved domains of the 16S–23S ITS rRNA region were identified based on comparative analysis utilizing knowledge of the basal clamps of the D1‐D1′, tRNA^Ile^, tRNA^Ala^, Box‐B, Box‐A, and D4 regions, as well as the V3 and D5 helices at the end of the ITS rRNA region. An alignment was constructed for the ITS rRNA region of the four species in the same genus (strains LEGE 16609, JJ‐SEL21‐B6, PCC 6802, and UPSI), and this alignment was used to calculate percent dissimilarity among these four species. M‐fold was used to construct the secondary structures with the force function used in the case of the D1‐D1′ helix so that the basal 3′ unilateral bulge was consistently shown. Within the alignment of the abovementioned four putative new species, a long sequence (65–68 nucleotides) was observed between the D3 region (UGGUUY) and the tRNA^Ile^. This highly unusual insert was checked in M‐fold, and a consistent helix formed that was 49–56 nucleotides long that we named the pre‐tRNA helix. The remaining strain under study (PCC 7502), a putative separate new genus, lacked the pre‐tRNA helix, but was the only one of the five species that possessed a V2 helix between the two tRNA genes, and this structure was determined for that strain. Secondary ITS rRNA region structures for all those strains were post‐edited in Adobe Illustrator.

Secondary structure diagrams of whole ribosomal operons were created in Adobe Illustrator for the cyanobacterial strains LEGE 16609, PCC 7502, *Pseudanabaena catenata* USMAC16, *Thalassoporum mexicanum* PCC 7367, and *Tumidithrix elongata* BACA0141, and are shown in Figures S1–, S5. Secondary structures of the 5S rRNA gene for all five putative new species, *Pseudanabaena catenata* USMAC16 and *Thalassoporum mexicanum* PCC 7367 were also created in Adobe Illustrator and likewise appear in Figure S6.

### Genome comparisons

All four genomes of one of the putative new genera (LEGE 16609, PCC 6802, JJ‐SEL21‐B6, and UPSI) were subjected to the calculation of average nucleotide identity (ANI) using FastANI (Jain et al., 2018) in the online server Galaxy version 1.3 (Blankenberg et al., 2014) with default parameters. The digital DNA:DNA hybridization (dDDH) was performed in silico using the online tool genome to genome distance calculator (GGDC) 3.0 (Meier‐Kolthoff et al., 2022). The average amino acid identity (AAI) was calculated using the command line interface for the EzAAI pipeline (Kim et al., 2021), with default parameters.

Additionally, genome size and GC content were recorded for all available representatives of Pseudanabaenales based on an NCBI Genome database search and our newly sequenced genomes (Figures S7, S8). Differences in these variables among the genus‐level clades were assessed using simple pairwise *t‐*tests.

### Taxonomic thresholds for molecular data

A number of established taxonomic thresholds based on DNA sequence data were utilized in making taxonomic decisions in the current study. For 16S rRNA gene sequence data, identities <98.7% have been considered evidence that strains belong to different species, whereas identities <94.5% have been considered evidence of different genera (Yarza et al., 2014). When using comparisons of percent dissimilarity in the ITS rRNA region to determine species, >7% dissimilarity has been considered strong evidence of species separation (Akagha et al., 2023; Cai et al., 2021; Hauerová et al., 2021; Mai et al., 2018; Mareš et al., 2019). Additionally, dissimilarities between 3% and 7% have also been used as evidence of lineage separation, even though interpretation at this level of difference has been considered somewhat ambiguous (Becerra‐Absalón et al., 2020; Bohunická et al., 2024; Pietrasiak et al., 2021). Although ITS rRNA region dissimilarities <3.0% have not been considered evidence of species separation on their own, in some cases, species with <3% ITS rRNA region dissimilarity have been considered different based upon other lines of evidence in polyphasic separation (Johansen et al., 2025).

In the current study, we further used taxonomic thresholds based on comparative genomics, namely ANI, AAI, and dDDH measures, as recommended by recent taxonomic reviews (Chun et al., 2018; Riesco & Trujillo, 2024). The threshold best established for delineation of bacterial species is 95%–96% ANI (Richter & Rosselló‐Móra, 2009), which corresponds to 95%–96% AAI (Konstantinidis & Tiedje, 2005) and to 70% dDDH, which has replaced the wet‐lab DDH previously used as a gold standard in prokaryotic taxonomy (Goris et al., 2007; Meier‐Kolthoff et al., 2013).

## RESULTS

### Taxonomic descriptions

#### *Portococcus* Strunecký, Mareš, J.R.Johansen et Vasconcelos gen. nov.

Diagnosis: Morphologically cryptic or semicryptic to *Synechococcus* and other *Synechococcus*‐like taxa. Separated by its multilocus phylogenetic position as a monophyletic clade of Pseudanabaenaceae and by the molecular structural synapomorphies in the 16S–23S ITS rRNA region including the presence of a pre‐tRNA helix, absence of a V2 helix between the tRNA genes, and an exceptionally long D5 helix (Figure S1).

Description: Cells solitary or in pairs before division. Single cells oval, usually with more or less straight middle section that might be partially constricted or bent, with rounded (hemispherical) ends after division, cells longer than wide, 1.4–3.1 μm wide, to 8.2 μm long, or occasionally spherical after division. Reproduction by binary fission, perpendicular to the longer axis of the cell, cells about two times longer than wide or more before division, not forming chains of divided cells. Thylakoids usually parietal from three to seven concentric layers, but might be also radial, with clearly delimited chromatoplasma and centroplasma. Freshwater or subaerophytic (epilithic), in temperate regions.

Etymology: *Porto*, a city in Portugal, home of the LEGE culture collection of cyanobacteria that provided source material for this generitype and *coccus*, a unicellular bacterial form. A coccal cyanobacterium from the Porto culture collection

#### Type species: *Portococcus lusitanicus* Strunecký, Mareš, Morais et Vasconselos sp. nov.

**FIGURE 1:**
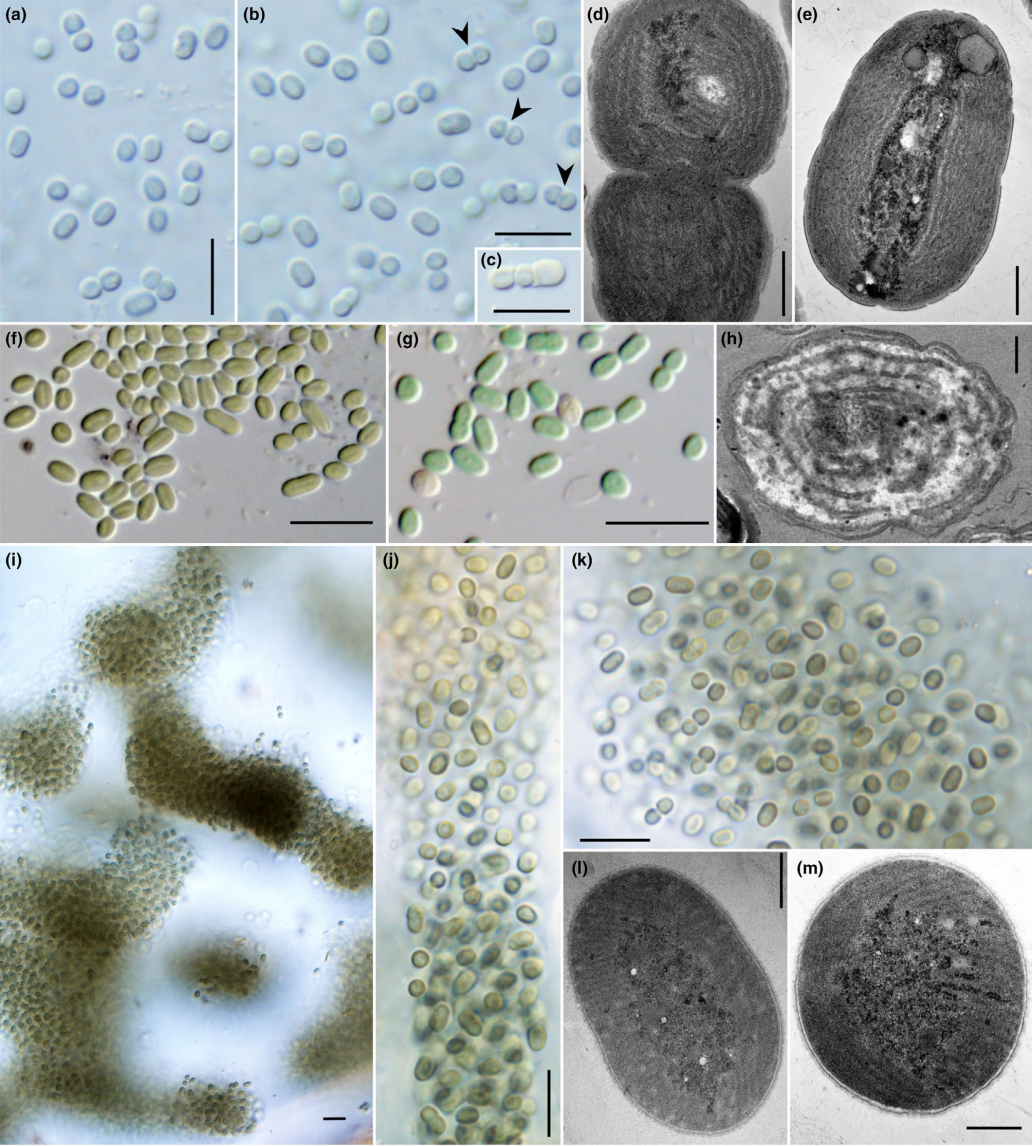
Morphology and ultrastructure of *Portococcus*. (a–e) *P. lusitanicus*; (f–h) *P. limneticus*; (i–m) *P. sanctae‐mariae*. Spherical cells are occasionally observed after binary fission in *Portococcus* (b, g). At the end of cell division, when the daughter cells are still attached, they reach a typical hemispherical shape (arrows) in *P. lusitanicus* whereas in other species they tend to be longer than wide at division (b). The freshwater strains lack sheath material (a, b, f, g) and contain parietally arranged thylakoids (d, e, h). The terrestrial strain of *P. sanctae‐mariae* forms irregular mucilaginous colonies (i–k) and exhibits a specific radial architecture of thylakoids with a prominent centroplasma (l–m).

Diagnosis: Microscopically very similar to *Portococcus sanctae‐mariae*, with a slightly lower average length:width ratio and cells very often distinctly shorter than wide and hemispherical at the end of cell division (when the new cell wall is finished, but before separation of the daughter cells), and with strictly parietal thylakoids. Further differs from *P. sanctae‐mariae* by its aquatic lifestyle. Cells are on average clearly shorter than in *P. limneticus* and *P. stanieri*. Separated by its multilocus phylogenetic position as a monophyletic clade of Pseudanabaenaceae. Also differing in ITS rRNA region structure by two unpaired adenine residues in the 5′ strand opposite the 3′ basal unilateral bulge of the D1‐D1′ helix, as well as minor structural differences in the pre‐tRNA and Box‐B helices (Figure 1a–e).

Description: Unicellular cyanobacteria with cells living solitary or in pairs of dividing cells, rarely in short rows of three to four cells after division, separated by hyaline cell walls (Figure 1b,c). Cells 2.3–3.1 μm (avg. 2.7 μm) wide and 2.1–4.5 μm (avg. 3.4 μm) long. The cells are shorter than wide (frequently L:W ratio ~ 0.8) and hemispherical at the end of division, later growing to be longer than wide (up to L:W ratio of 1.7), straight, strictly cylindrical with rounded ends. The cell content is blue–green, later with a brownish tint, usually finely granular, with a delimited centroplasma and chromatoplasma. Gas vesicles not observed. Thylakoids parietal with three to seven concentric layers (Figure 1d,e).

Holotype here designated: CBFS A–264, dried material of the strain *Portococcus lusitanicus* LEGE 16609 (metabolically inactive), deposited in the herbarium collection of the University of South Bohemia. Original material collected by Tiago Guerra in a stagnant pool of water, part of a freshwater stream at Barrancos dos Pisões (37.338° N, 8.573° W), Serra de Monchique, Portugal (type locality).

Reference strain: *Portococcus lusitanicus* LEGE 16609, isolated by Maria Fátima Santos

Etymology: *lusitanicus*, of Lusitania, the Roman name for much of Portugal today, where the new *Portococcus* species was isolated

Taxonomic notes: Morphology known strictly from culture.

#### *Portococcus limneticus* Strunecký, Jezberová et Mareš sp. nov.

Diagnosis: Microscopically similar to *Portococcus lusitanicus* and *P. sanctae‐mariae* but the cells are on average longer (never shorter than 3 μm). The cell width is uniform among these three species. Cells on average distinctly shorter and wider than in *P. stanieri*, with yellow‐brown color in older cells. Defined by its multilocus phylogenetic position as a monophyletic clade of Pseudanabaenaceae. Most similar to *P. sanctae‐mariae* in ITS rRNA structure and sequence but differing in the structure of Box‐B and V3 helices from that taxon. Differing from other species in position and size of mismatched‐nucleotide bilateral openings (Figure 1f–h).

Description: Unicellular cyanobacteria with cells living solitary or in pairs of dividing cells, separated by hyaline cell walls (Figure 1f,g). Cells 2.1–3.1 μm (avg. 2.6 μm) wide and 3.5–5.5 μm (avg. 4.6 μm) long. The cells are always longer than wide after division (L:W ratio ~ 1.2), later growing to 2.3 times longer than wide, oval to cylindrical with rounded ends. The cell content is blue–green, later with a yellow‐brownish tint, homogenous to finely granular, with a delimited centroplasma and chromatoplasma. Gas vesicles not observed. Thylakoids parietal with three to seven concentric layers (Figure 1h).

Holotype here designated: CBFS A–265, dried material of the strain *Portococcus limneticus* JJ‐SEL21‐B6 (metabolically inactive), deposited in the herbarium collection of the University of South Bohemia. Original material collected by Jitka Jezberová in open water, 0.5 m depth, of a mesotrophic post‐mining lake, Lake Senftenberg (51.501° N 14.049° E), Senftenberg, Germany (type locality).

Reference strain: *Portococcus limneticus* JJ‐SEL21‐B6, isolated by Jitka Jezberová

Etymology: *limneticus* (L.), referring to the limnetic habitat of the organism

Taxonomic notes: Morphology known strictly from culture.

#### *Portococcus sanctae‐mariae* Mareš, Čapková, Toledo‐Lemus et Strunecký sp. nov.

Diagnosis: Microscopically similar to both *Portococcus limneticus* and *P. lusitanicus*, but subaerophytic rather than aquatic, forming large mucilaginous colonies with intense dark‐brown pigmentation. Defined by its multilocus phylogenetic position as a monophyletic clade of Pseudanabaenaceae. Most similar to *P. limneticus* in its ITS rRNA structure and sequence but differing in the structure of Box‐B helix from that taxon. Differing from other species in position and size of mismatched‐nucleotide bilateral openings (Figure 1i–m).

Description: Thallus thin, smooth, black, firmly attached to a solid substrate. Unicellular cyanobacteria with cells living solitary or in pairs of dividing cells, separated by hyaline cell walls, forming large irregular colonies of densely packed cells in transparent and diffluent mucilage (Figure 1i–k). Cells 2.0–3.1 μm (avg. 2.6 μm) wide and 2.2–4.8 μm (avg. 3.5 μm) long. The cells are isodiametric or slightly shorter or longer than wide after division (L:W ratio ~ 1), later growing to 1.9 times longer than wide, oval to cylindrical with rounded ends. The cell content is blue–green to dark gray‐brown, homogenous to finely granular, with a delimited centroplasma and chromatoplasma. Gas vesicles not observed. Thylakoids are parietally to radially organized, absent in the central part of the cell (Figure 1l,m).

Holotype here designated: CBFS A–268, dried material of the strain *Portococcus sanctae‐mariae* UPSI (metabolically inactive), deposited in the herbarium collection of the University of South Bohemia. Original material collected by Kateřina Čapková from an epilithic biofilm growing on a wet rock in the entrance of an alpine limestone cave with low irradiance and high moisture, Wetterloch cave (47.771° N, 13.441° E), 1518 m a.s.l., Schafberg, near to St. Wolfgang, Austria (type locality).

Reference strain: *Portococcus sanctae‐mariae* UPSI, isolated by Kateřina Čapková

Etymology: The name refers to the statue of Holy Virgin Mary situated at the Wetterloch cave entrance, just above the rock from which the species is described.

#### *Portococcus stanieri* Strunecký, Mareš et J.R.Johansen sp. nov.

**FIGURE 2:**
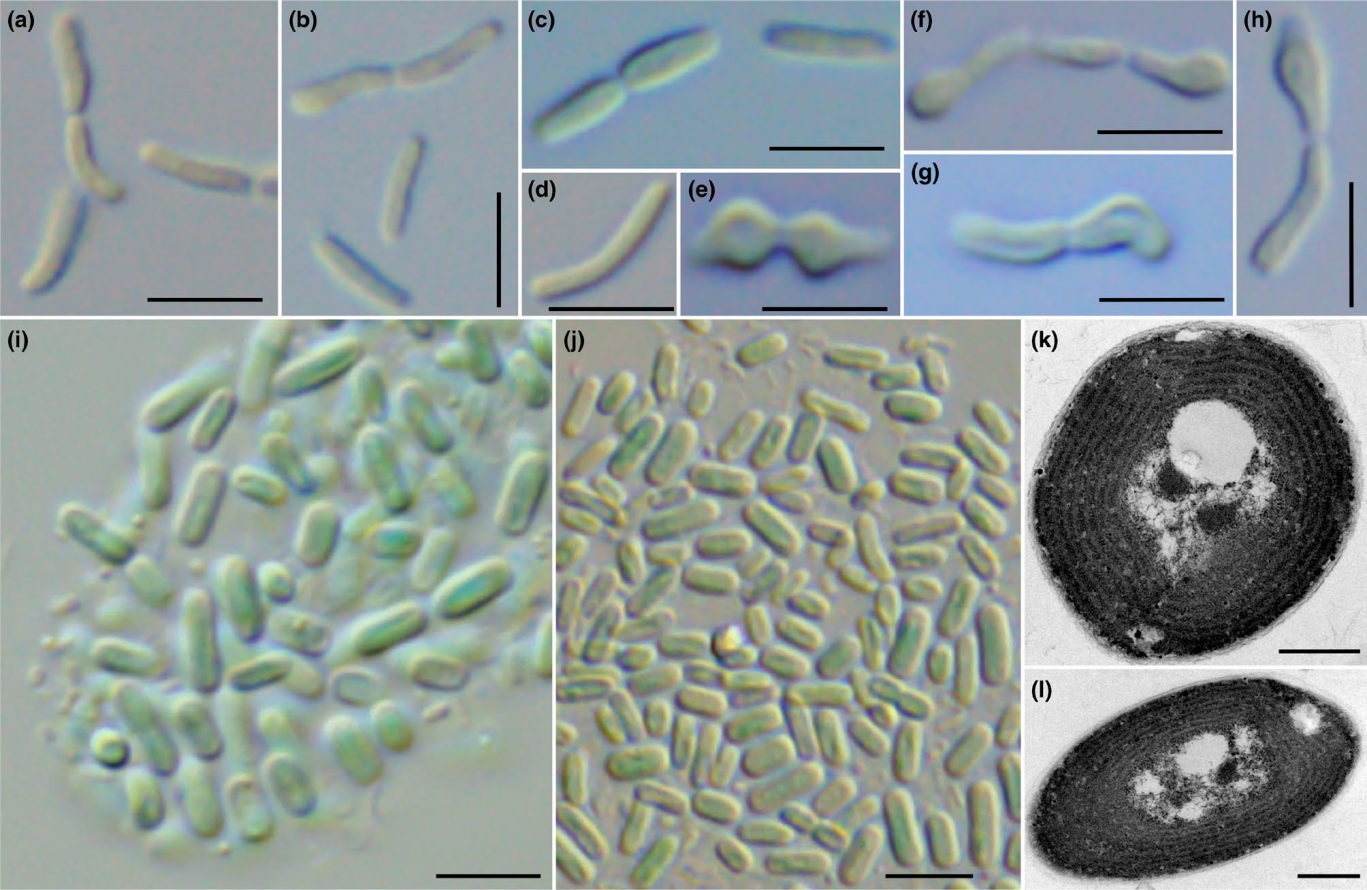
Morphology of *Portococcus stanieri* and *Pseudanabaenococcus habilitatus*. (a–h) *P. stanieri*; (i–l) *Ps. habilitatus*. Frequent occurrence of involution cells was observed in the strain PCC 6802 (e–h). Spherical cells were not observed in *Pseudanabaenococcus* (i, j).

Diagnosis: Microscopically distinct from other known species of the genus by its average significantly longer and narrower cells that are two to five times longer than wide. The cells are blue green but never brownish as in all the other species. Molecularly differing from other species in the genus by the presence of a notably longer V3 helix (Figure 2a–h).

Description: Unicellular cyanobacteria with cells living solitary or in pairs of dividing cells separated by hyaline cell walls (Figure 2a–d). Cells 1.4–2.2 μm (avg. 1.7 μm) wide and 3.1–8.2 μm (avg. 5.7 μm) long. The cells are distinctly longer than wide (L:W ratio ~2) and cylindrical after division, later growing to five times longer than wide, straight or arcuated, with rounded ends. The cell content is blue–green, homogenous. Gas vesicles not observed in this study but originally reported (Guglielmi & Cohen‐Bazire, 1984). Thylakoids parietal with three to seven concentric layers (Guglielmi & Cohen‐Bazire, 1984). Capable of anaerobic N_2_ fixation.

Holotype here designated: CBFS A–266, dried material of the strain *Portococcus stanieri* PCC 6802 (metabolically inactive), deposited in the herbarium collection of the University of South Bohemia. Original material collected in 1968 by A. Neilson from a freshwater pond (37.876° N, 122.240° W) at the University of California, Berkeley, California, United States (type locality).

Reference strain: *Portococcus stanieri* PCC 6802, isolated by A. Neilson as “*Synechococcus* sp. B5”

Etymology: Named in honor of Prof. R.Y. Stanier, a famous cyanobacteriologist, one of the leaders behind the development of the PCC culture collection, who also first recognized that the strain PCC 6802 is closely related to the genus *Pseudanabaena*.

Taxonomic notes: Morphology of the species is known strictly from culture. In the subculture examined within this study, abundant malformation of cells (so‐called involution cells) was observed (Figure 2e–h), which could have been acquired during long‐term laboratory cultivation of the strain since 1968.

#### *Pseudanabaenococcus* Strunecký gen. nov.

**FIGURE 3:**
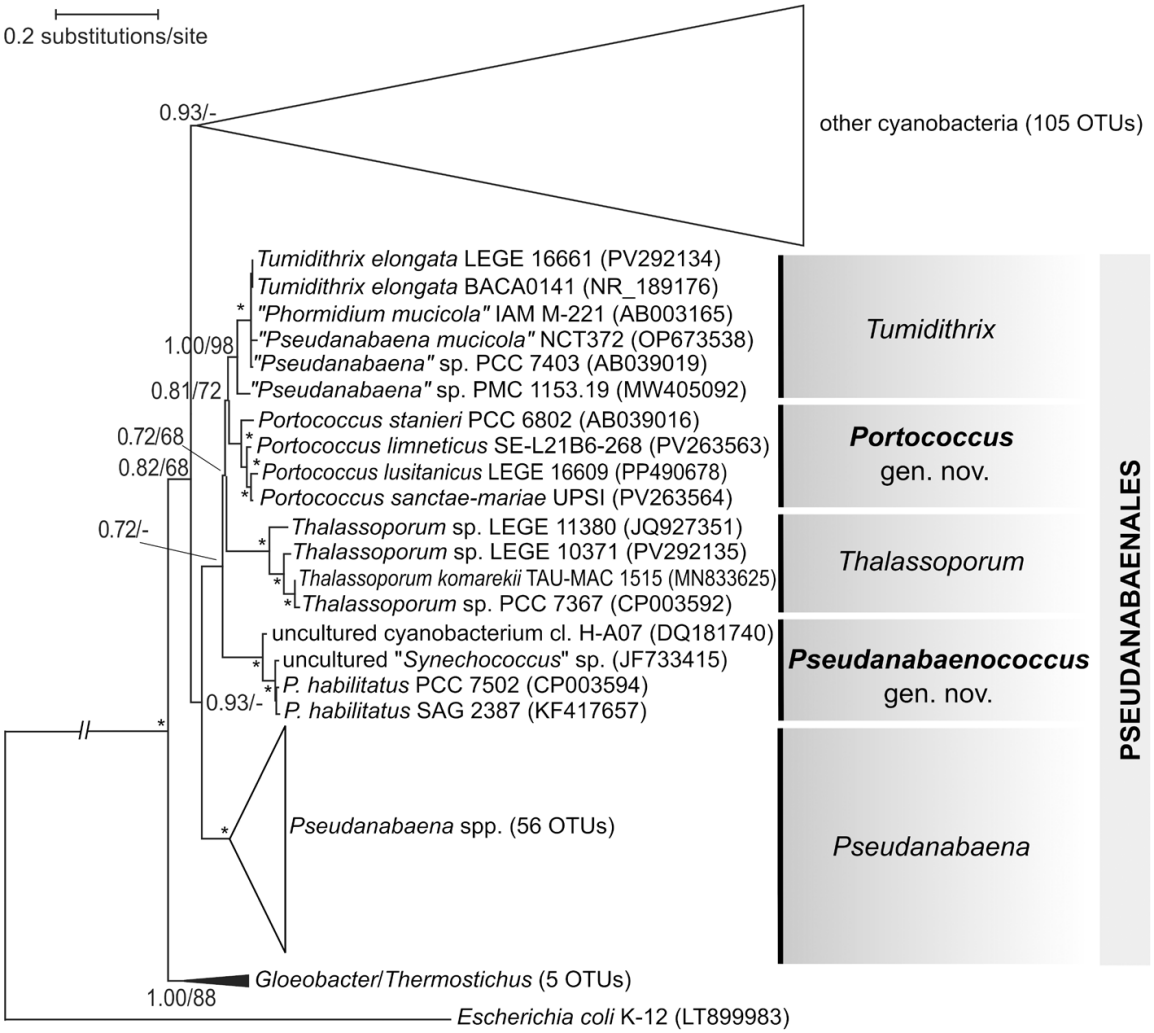
16S rRNA phylogenetic tree (Bayesian inference) of cyanobacteria, showing the clustering of genera within the order Pseudanabaenales, including two new genera, *Portococcus* and *Pseudanabaenococcus*, resolved as monophyletic sub‐clades. Branch supports ≥50% are shown at the nodes; asterisk indicates the value of 1/100 (BI posterior probability/ML bootstrap).

**FIGURE 4:**
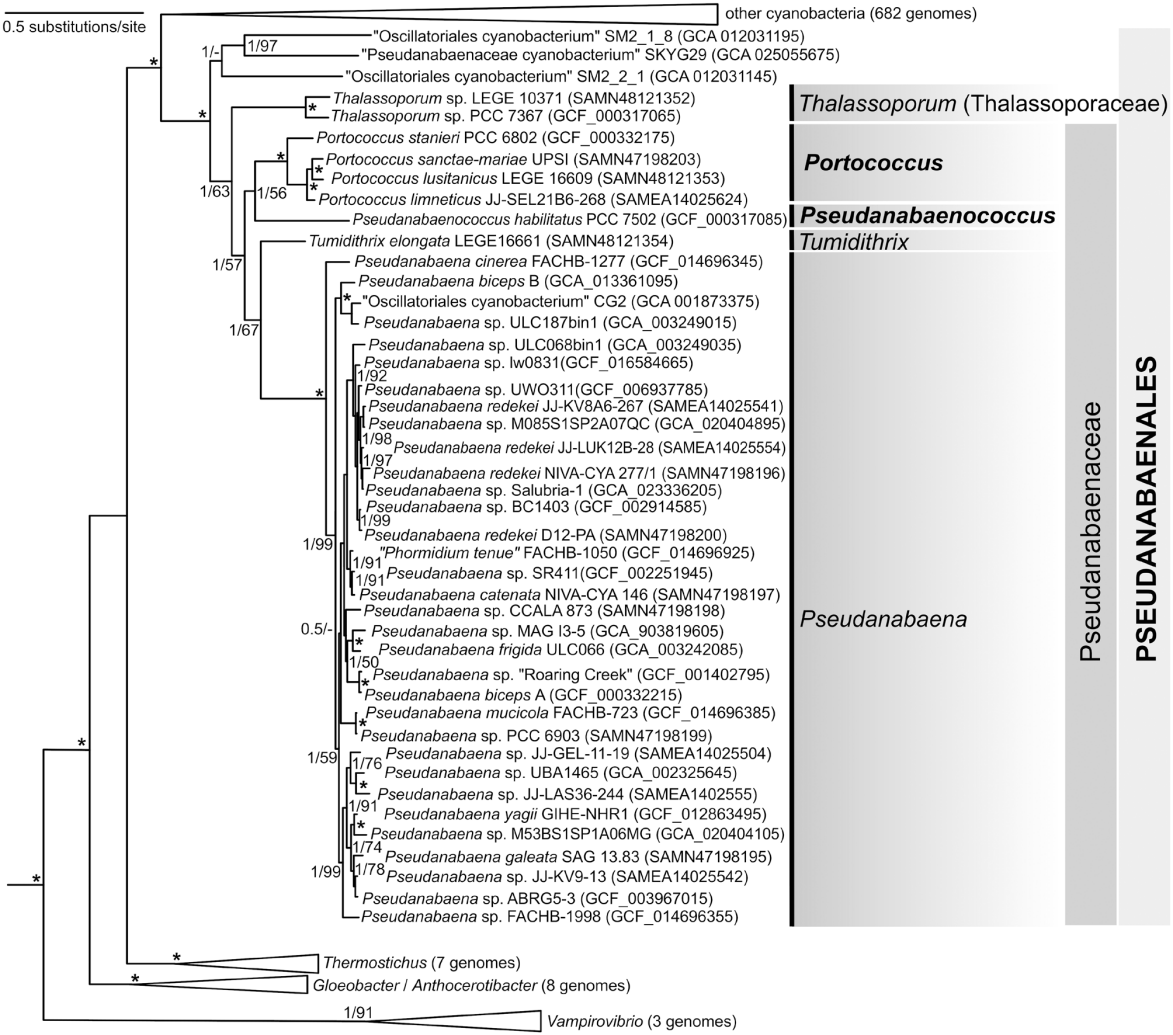
Phylogenomic tree (maximum likelihood, 120 conserved bacterial proteins) of cyanobacteria, showing the clustering of families and genera within the order Pseudanabaenales. The two newly described genera, *Portococcus* and *Pseudanabaenococcus*, are grouped in a monophyletic unicellular sub‐lineage within the family Pseudanabaenaceae. Branch supports ≥50% are shown at the nodes; asterisk indicates the value of 1/100 (BI posterior probability/ML bootstrap).

Diagnosis: Morphologically cryptic with *Synechococcus* and *Portococcus*, however forms a deeply phylogenetically separated lineage within Pseudanabaenales (Figures 3, 4). The genome‐sequenced strain PCC 7502 has a significantly smaller genome size and lower GC content compared to the neighboring genera in Pseudanabaenales (Figures S7, S8). The ITS rRNA region secondary structure differing from *Portococcus* in the possession of a V2 helix and absence of the pre‐tRNA helix (Figures S1, S2). Differing from *Pseudanabaena* and *Thalassoporum* in possession of a branching terminus in the ITS rRNA region producing both V3 and D5 helices (Figures S2, S3). The 5S rRNA molecule begins at the 5′ end with 5′‐GCUU‐3′, differing from *Portococcus* (5′‐UCCU‐3′), *Thalassoporum* (5′‐UCCU‐3′) (Figures S1, S2, S4), and most other cyanobacteria (5′‐UCCU‐3′).

Description: Unicellular cyanobacteria, cells living solitary or in agglomerations formed by cylindrical cells with rounded or filleted ends (Figure 2i,j), longer than wide, 1.5–3.0 μm wide, to 7.0 μm in length, strictly cylindrical or slightly arcuated, with sometimes visible sheaths (1000× magnification). Reproduction by binary fission, perpendicular to the long axis of the cell in subsequent generations, cells are more than three times longer than wide before division, not forming chains of divided cells. Thylakoids parietal (Figure 2k,l). Freshwater epilithic, or epiphytic.

Etymology: Referring to *Pseudanabaena*, a related genus of simple filamentous cyanobacteria, and *coccus* (L.), unicellular bacterial form. A unicellular cyanobacterium related to *Pseudanabaena*

#### Type species: *Pseudanabaenococcus habilitatus* Strunecký sp. nov.

Description: Unicellular cyanobacteria with cells 2.0 μm wide (in the range from 1.5 μm to 2.7 μm) and 4.5 μm long (in the range from 2.9 μm to 6.6 μm) (Figure 2i,j). Cells solitary, or in irregular agglomerations, blue–green or green with rounded ends, longer than wide, straight, strictly cylindrical, or slightly arcuated, with visible but thin and colorless sheaths. Reproduction by binary fission, perpendicular to the long axis of the cell in subsequent generations, symmetrical or slightly asymmetrical, daughter cells separating soon after division. Gas vesicles have not been observed. Thylakoids parietal, with five to seven concentric layers and a delimited centroplasma (Figure 2k,l).

Holotype here designated: CBFS A–267, dried material of the strain *Pseudanabaenococcus habilitatus* SAG 2387 (metabolically inactive), deposited in the herbarium collection of the University of South Bohemia. Original material collected by A. HodaČová from a tufa forming biofilm in a highland freshwater stream, Westerhöfer Creek (51.750° N, 10.083° E), near Westerhof, Germany (type locality).

Reference strain: *Pseudanabaenococcus habilitatus* SAG 2387, isolated by Alena HodaČová as “*Synechococcus* sp. RKC4.” Another prominent strain belonging to this species is PCC 7502 isolated by R. Rippka (as “*Synechococcus* sp.”) from a sphagnum bog, near Kastanienbaum, Vierwaldstattersee, Switzerland, 1972. The latter strain was published as *Leptovivax bogii* in Walter et al. (2017) based on a genomic sequence of PCC 7502 but did not meet the nomenclatural and orthographic requirements of the *International Code of Nomenclature for algae, fungi, and plants* (ICN, see Turland et al., 2025) or the *International Code of Nomenclature for Prokaryotes* (ICNP, see Oren et al., 2022). *Leptovivax bogii* consequently has no nomenclatural status (see discussion section for further comments).

Etymology: *habilitatus*, (L.), enabled, enabling, enables; in honor of those who have devoted their academic careers to the study of cyanobacteria

### Phylogenetic and bioinformatic analyses

Multilocus and 16S rRNA gene phylogenies yielded almost identical groupings of strains at the genus level in Pseudanabaenales although the topology differed somewhat at the deeper nodes (Figures 3, 4, S9, S10). The most striking difference between the two trees was the separation of the families Thalassoporaceae and Pseudanabaenaceae in the genomic tree, which was not supported in the 16S rRNA gene tree. The 16S rRNA gene tree, however, in general, mostly lacked statistical support at the family and higher levels (Figure 3).

Within the phylogenomic cluster corresponding to Pseudanabaenaceae, besides the large clade of strains belonging clearly to various species of *Pseudanabaena* sensu stricto, three other discrete clades were resolved. The first of them, the sister clade to *Pseudanabaena*, was represented by a single strain, LEGE 16661, and corresponded to the recently described genus *Tumidithrix*. Closer to the root, but still within Pseudanabaenaceae, two distinct clades composed solely of coccoid strains were inferred. The better‐sampled genus‐level clade, herein described as *Portococcus*, contained four non‐filamentous strains, including a classical strain from the Pasteur Culture Collection (“*Pseudanabaena*” sp. 6802) and three strains of “*Synechococcus*” sp. (JJ‐SEL21B6, LEGE 16609, and UPSI) isolated in the framework of this study. The second clade containing “*Synechococcus*” sp. strains SAG 2387 and PCC 7502 had already been suggested to be a separate genus by Strunecký et al. (2023). As described above, this clade has been formally described as *Pseudanabaenococcus*. The phylogenomic lineages of the two new coccoid genera (Figure 4) were further supported in the better‐sampled 16S rRNA gene tree (Figure 3) as well as by the 16S rRNA gene distance to neighboring taxa higher than 5% (Table 2).

**TABLE 2:** Percent similarity matrix (100 – (p‐distance*100)) of partial 16S rRNA gene sequence (1072 bp compared) for *Portococcus stanieri*, *P. limneticus*, *P. lusitanicus*, *P. sanctae‐mariae*, and *Pseudanabaenococcus habilitatus*, compared to other species within Pseudanabaenales and representatives of selected neighboring phylogenetic clusters. Shaded boxes represent strain pairs belonging to the same genera.

Strain #		1	2	3	4	5	6	7	8	9	10	11	12	13	14	15	16	17	18	19	20	21	22	23
1	*Portococcus stanieri* PCC 6802																							
2	*Portococcus limneticus* JJ‐SEL21B6	97.1																						
3	*Portococcus lusitanicus* LEGE 16609	97.2	98.4																					
4	*Portococcus sanctae‐mariae* UPSI	97.0	98.6	98.9																				
5	*Tumidithrix elongata* PCC 7403	94.8	95.3	94.4	94.9																			
6	*Pseudanabaenococcus habilitatu*s SAG 2387	91.8	91.6	91.5	91.6	92.8																		
7	*Pseudanabaenococcus habilitatus* PCC 7502	91.7	91.9	92.0	92.1	92.9	99.2																	
8	*Pseudanabaena catenata* SAG 254.80	91.7	92.7	92.4	92.1	91.9	90.6	90.9																
9	*Pseudanabaena minima* GSEPSE‐2005C	91.3	92.4	92.2	91.9	91.8	90.5	90.8	98.9															
10	*Limnothrix* sp. Sai002	91.3	92.3	92.1	91.8	92.0	90.7	91.0	99.0	98.6														
11	*Pseudanabaena* sp. PCC 6903	91.8	92.6	92.3	92.0	92.0	90.1	90.4	99.0	98.4	98.9													
12	*Pseudanabaena* sp. CCALA 873	91.0	91.9	91.7	91.4	91.8	90.7	91.0	98.0	98.6	98.5	98.3												
13	*Pseudanabaena galeata* SAG 13.83	91.5	92.2	92.0	91.7	91.8	90.5	90.8	98.4	99.0	98.7	98.5	98.7											
14	*Pseudanabaena cinerea* CHAB2916	91.9	92.7	92.7	92.4	92.0	90.2	90.7	98.0	97.5	98.2	98.1	97.2	98.0										
15	*Pseudanabaena limnetica* CHAB792	90.3	91.5	91.4	91.1	91.4	89.9	90.1	94.2	94.8	94.6	94.5	94.7	95.3	95.2									
16	*Pseudanabaena* sp. PCC 7367	92.0	92.2	91.9	91.9	91.6	90.0	89.9	89.2	88.7	88.9	89.2	88.9	89.2	89.5	88.0								
17	*Thalassoporum komareki* TAU‐MAC 1515	92.2	92.4	92.0	92.1	91.8	90.2	90.1	89.2	88.8	88.8	89.2	88.9	89.1	89.4	87.9	99.3							
18	*Thalassoporum* sp. LEGE 10371	92.9	92.9	92.4	92.3	91.8	90.2	90.1	89.1	88.6	88.6	89.1	88.6	88.9	89.1	87.9	97.5	97.8						
19	*Thalassoporum* sp. LEGE 11380	92.2	93.4	92.5	92.6	92.8	88.9	88.8	90.2	89.7	89.9	90.0	89.8	89.9	90.1	89.1	95.6	95.7	95.9					
20	*Limnothrix redekei* 165a	87.9	87.5	87.6	87.8	88.4	89.1	89.2	89.3	89.1	89.5	89.5	89.3	89.1	89.0	90.1	87.7	87.9	87.5	87.3				
21	*Thermostichus* sp. JA‐2‐3B'a	88.0	88.5	88.0	88.1	88.3	86.2	86.7	88.8	87.8	88.4	89.1	88.2	88.0	88.4	87.9	88.8	88.7	87.8	88.4	86.6			
22	*Gloeobacter violaceus* PCC 9601	89.0	88.7	88.4	88.6	89.2	87.2	87.2	89.3	88.4	89.0	89.5	88.4	88.8	88.6	87.5	88.8	89.0	88.4	88.8	87.4	90.4		
23	*Leptolyngbya boryana* UTEX B 488	86.8	87.3	87.3	87.2	87.8	87.7	87.9	88.2	88.1	88.2	88.0	88.4	88.1	88.6	89.0	88.1	88.3	87.6	87.1	89.0	86.9	86.9	
24	*Geitlerinema* cf. *pseudacutissimum* CCALA 142	88.1	88.4	88.4	88.5	87.3	88.4	88.3	87.8	87.7	87.7	87.9	87.9	87.8	87.4	86.9	88.1	88.2	89.0	88.0	87.5	85.7	86.5	86.2

Presently, *Pseudanabaenococcus* is rather homogenous from the perspective of the 16S rRNA gene similarity (99.2%, Table 2), supporting the recognition of only a single species. Conversely, *Portococcus* strains exhibited a considerably higher divergence within the 16S rRNA gene (pairwise sequence identity of 97.0%–98.9%, Table 2), ITS rRNA region (pairwise p‐distances ranging 6.8%–27.0%, Table 3), genome‐to‐genome ANI values lower than 87%, AAI values <89%, and dDDH values <38% (Table 4). These results consistently indicate that each of the four *Portococcus* strains represented a separate species.

**TABLE 3:** Percent dissimilarity among *Portococcus* species. Values >7.0% dissimilarity are considered strong evidence of separate species lineages; values of 4.0%–7.0% dissimilarity typically indicate separate species; values <3.0% dissimilarity generally indicate strains belong to the same species lineage.

	Strain #	1	2	3
*Portococcus lusitanicus* LEGE 16609	1			
*Portococcus limneticus* JJ‐SEL21B6	2	8.3%		
*Portococcus sanctae‐mariae* UPSI‐S4	3	8.4%	6.8%	
*Portococcus stanieri* PCC 6802	4	26.4%	24.5%	27.0%

**TABLE 4:** Genome‐to‐genome similarity measures among the *Portococcus* strains.

	Strain #		1	2	3
ANI	1	*Portococcus lusitanicus* LEGE 16609			
	2	*Portococcus limneticus* JJ‐SEL21B6	86.0%		
	3	*Portococcus sanctae‐mariae* UPSI	86.1%	86.3%	
	4	*Portococcus stanieri* PCC 6802	78.1%	79.1%	78.1%
AAI	1	*Portococcus lusitanicus* LEGE 16609			
	2	*Portococcus limneticus* JJ‐SEL21B6	88.1%		
	3	*Portococcus sanctae‐mariae* UPSI	88.1%	88.0%	
	4	*Portococcus stanieri* PCC 6802	77.9	78.9%	78.2
dDDH	1	*Portococcus lusitanicus* LEGE 16609			
	2	*Portococcus limneticus* JJ‐SEL21B6	34.9%		
	3	*Portococcus sanctae‐mariae* UPSI	37.2%	34.9%	
	4	*Portococcus stanieri* PCC 6802	17.1%	18.3%	16.9%

Abbreviations: AAI, average amino‐acid identity; ANI, average nucleotide identity; dDDH, digital DNA:DNA hybridization.

**FIGURE 5:**
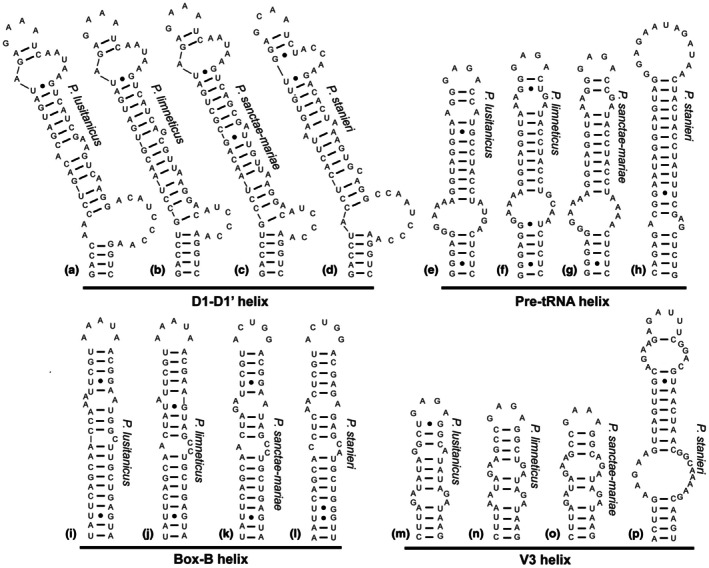
Predicted ITS rRNA region secondary structures within *Portococcus* species. Note the similarity in structure in helices of *P. limneticus* and *P. sanctae‐mariae*, while the helices for *P. lusitanicus* and *P. stanieri* are distinctly different. *Portococcus* lacked the V2 in all strains.

An ITS rRNA region analysis of the *Portococcus* strains showed that three of the proposed species, *P. lusitanicus*, *P. limneticus*, and *P. sanctae‐mariae*, were fairly similar in secondary structure in all four conserved helices, although differences in sequence and minor differences in structure were observed in every comparison (Figure 5). *Portococcus limneticus* and *P. sanctae‐mariae* were the most similar pair of species and also had the lowest ITS rRNA region percent dissimilarity (6.8%). The combination of ITS rRNA region dissimilarity and differences in the helix structures, along with the 16S rRNA gene percent similarity and ANI values provided good evidence for the separation of these two species. *Porotcoccus lusitanicus* had more evident differences in the ITS rRNA region structure (see Figure 5a–c,m–o).

*Portococcus stanieri* was distinctly different in three of the ITS rRNA region helix structures (D1‐D1′, pre‐tRNA, and V3). Furthermore, its ITS rRNA region percent dissimilarity was >24% in all three comparisons with the other species. This level of difference could be used as justification for recognizing this species as a third novel genus. However, the presence of the pre‐tRNA helix was a strong synapomorphy that tied these four species together, and the 16S rRNA phylogeny and phylogenomic analysis both showed that all four species are in the same clade. Upon discovery of more species in this clade, an additional genus might be recognized in the future.

More ITS rRNA region peculiarities were observed. In all cases with which we are familiar, the D2 has ended with 5′‐CAAACU‐3′, which has been conserved due to the pairing with the ribosomal leader region. This sequence was preserved in *Pseudanabaenacoccus* but absent in all four *Portococcus* species (Figures S1, S2). The D5 region (which pairs with the 23S–5S ITS rRNA region) was atypically long in all five taxa (21–42 nucleotides; Figures S1, S2), further evidence that this is a distinctive clade of cyanobacteria. Finally, comparison with the generitype of the sister genus *Pseudanabaena, Ps. catenata*, showed that *Pseudanabaena* lacks a branching structure at the 16S–23S ITS rRNA region terminus, such that only an exceptionally long D4 helix can be identified (Figure S3).

Comparisons of the 5S rRNA structure among six abovementioned species of Pseudanabaenaceae were made and supported the possible placement of *Pseudanabaenococcus* in a phylogenetically close relationship with *Pseudanabaena*, with *Portococcus* occupying a more distant place from both taxa. This finding was in congruence with the topology of the 16S rRNA gene tree (Figure 3) but not the phylogenomic tree (Figure 4). Particularly of note was the beginning of the 5S rRNA gene in *Pseudanabaenococcus* and *Pseudanabaena*, which shared the sequence 5′‐GCUU‐3′. All *Portococcus* species and *Thalassoporum mexicanum* started with 5′‐UCCU‐3′ (Figure S6), which is the more common starting sequence observed by us thus far in cyanobacteria. These figures required assembly using comparative analysis and could not be generated in Mfold due to the high number of non‐canonical base pairings (Figure S6).

### Morphology and ultrastructure

The representatives of the proposed genus *Portococcus* are unicellular and have oval shaped cells, in which the cell length:width ratio is usually lower than in typical *Synechococcus*. As an exception to this, *P. stanieri* (strain PCC 6802) has slightly thinner and longer cells than the other representatives, although pictures of clearly spherical cells after division were reported by Guglielmi and Cohen‐Bazire (1984) in strain PCC 6802. Similarly, spherical cells are occasionally present in all *Portococcus* strains, especially *P. lusitanicus* LEGE 16609 (Figure 1a,b), although their frequency after completed cell division appears to be rather low. Interestingly, species of *Portococcus* have frequently formed constrictions and hyaline cell walls at the site of cell division (Figures 1b,c,k, 2b,c), resembling the morphology of their filamentous Pseudanabaenalean relatives. Morphological variation in the described species may therefore have resulted from physical and chemical conditions in both culture and nature and should be addressed in future studies.

*Pseudanabaenococcus habilitatus* is a unicellular cyanobacterium with cells ~ 2.0 μm wide and 5 μm long (Figure 2i,j). It is rather indistinguishable from *Portococcus* based on morphology. In contrast to *Portococcus*, we have not observed the occurrence of spherical cells after division in the studied strain.

The ultrastructural observations of aquatic strains belonging both to *Portococcus* and *Pseudanabaenococcus* confirmed the presence of parietal thylakoids, typically containing four to seven layers of thylakoid membranes (Figures 1d,e,h, 2k,l). However, the subaerophytic strain isolated from a shaded cave entrance, *Portococcus sanctae‐mariae* UPSI, unexpectedly exhibited a radial arrangement of thylakoids, although still with a marked centroplasma (Figure 1l,m). Analyses of TEM images did not reveal the presence of gas vesicles, despite the fact that their existence in some strains cannot be completely ruled out, as Guglielmi and Cohen‐Bazire (1984) identified such structures in *P. stanieri* strain PCC 6802, although the designation of gas vesicles in those published images was not entirely unambiguous.

### Ecology

The strains were isolated from different environments, further supporting the separation of the proposed species. The oldest known strain *Portococcus stanierii* PCC 6802 was isolated from a pond in California, United States; *P. limneticus* originated from a post‐mining lake in Germany; *P. lusitanicus* LEGE 16609 was sampled in a mountain brook in Portugal; and *P. sanctae‐mariae* UPSI grew at a calcareous alpine cave rock portal at Schafberg, Austria. Surprisingly, no additional sequences belonging to *Portococcus* were available in GenBank, which left the resolution of its ecological distribution dependent on future discoveries.

The two available *Pseudanabaenococcus habilitatus* strains were obtained from submersed biofilms in Central European mountains. Closely affiliated environmental sequences from a microbial mat in Lake Heart, East Antarctica (DQ181740; Taton et al., 2006), and from an aquatic insect larval microbiome from North America (JF733415; Tang et al., 2012) suggest a probable wider geographical occurrence of the genus in submersed biofilms across the globe.

## DISCUSSION

Despite their ubiquity, members of the cyanobacterial order Pseudanabaenales have not been in the spotlight of cyanobacterial research for a long time. Several factors may have contributed to this neglect. Although the morphology and ultrastructure of *Pseudanabaena* and its relatives are distinctive (Guglielmi & Cohen‐Bazire, 1984), they tend to be rather uniform across the order, which has long prevented the understanding of their taxonomic relationships. Pseudanabaenales strains are often outcompeted and overgrown by faster growing cyanobacteria from orders such as Leptolyngbyales, Oscillatoriales, or Coleofasciculales, further complicating their isolation, study, and characterization (Strunecký et al., 2020). As a result, many cryptic species classified under the genus *Pseudanabaena* were often identified incidentally during ecological or metabolomic studies (Aleksovski et al., 2024; Tuji & Niiyama, 2018). Such recently acknowledged cryptic diversity in Pseudanabaenales warranted the establishment of two new genera: *Thalassoporum* and *Tumidithrix* (Konstantinou et al., 2021; Luz et al., 2023). The only notable morphological distinction in *Tumidithrix* was the presence of swollen cells, a feature that may be an artifact of cultivation conditions and requires further investigation to determine its biological relevance (Luz et al., 2023). *Thalassoporum* is morphologically cryptic but differs from *Pseudanabaena* by its marine origin. This intriguing morphological stability observed in Pseudanabaenales (Aleksovski et al., 2024) suggests that their size, shape, and fundamental metabolic traits are highly adaptive in the ecosystems where they occur. The enduring presence of these forms underscores their evolutionary success and highlights the remarkable resilience of their core biological characteristics, which have likely remained unchanged over vast evolutionary timescales (Schirrmeister et al., 2015). Considering their long evolutionary history, the recognition of many more cryptic taxa within Pseudanabaenales should be anticipated in the future.

Taxonomic assignments of unicellular cyanobacteria classified under the genus epithet “*Synechococcus*” have long been unclear. Historically, these organisms were considered to belong to the order Synechococcales (Korelusová et al., 2009). Only recently has careful polyphasic analysis of individual *Synechococcus*‐like strains determined the phylogenetic position of *Synechococcus* sensu stricto (Komárek et al., 2020). Although this taxonomic revision has led to the reclassification of several basal *Synechococcus*‐like strains into *Thermostichus*, *Aegeococcus*, and *Parathermosynechococcus* (Tang et al., 2023), other strains long known from cyanobacterial culture collections have continued to be mislabeled. Among these unresolved strains, “*Pseudanabaena*” sp. PCC 6802 and “*Synechococcus*” sp. SAG 2387 occupied long branches in 16S rRNA gene trees with their taxonomic assignment remaining unclear (Strunecký et al., 2023).

Given that the explanatory power of the 16S rRNA gene is limited in phylogenies above the genus level (Mareš, 2018) and that evolutionary histories of the rRNA operons can be complex (Johansen et al., 2017; Villanueva et al., 2024), in the current study we employed whole‐genome sequencing to resolve the taxonomic position of these strains. Our genomic phylogeny (Figure 4) supported the monophyly of Pseudanabaenales, consisting of two families, Pseudanabaenaceae and Thalassoporaceae (Strunecký et al., 2023). Interestingly, the unicellular strains formed a distinct lineage within this otherwise filamentous order. Although the taxon sampling in the genomic tree was still limited, its topology supported the hypothesis that the coccoid clade consisting of the new genera *Portococcus* and *Pseudanabaenococcus* likely evolved as a result of a secondary loss of multicellularity within the family Pseudanabaenaceae. Considering that the common ancestor of Pseudanabaenales is believed to represent the first filamentous form in cyanobacterial evolution (Schirrmeister et al., 2011), the new addition of whole‐genome sequences to the NCBI database offers an excellent opportunity to study the genetic and physiological fundaments of prokaryotic multicellularity. The shape of cells and their organization tend to remain uniform within individual strains of Pseudanabaenales. However, the number of cells within a filament can sometimes vary with culture conditions. Notably, in the unicellular *Portococcus*, two‐ to three‐celled “filaments” have been occasionally observed (Guglielmi & Cohen‐Bazire, 1984, Figure 1c), depending on the physiological state. This suggests that the distinction between filamentous and coccal forms in the basal filamentous cyanobacteria may be less rigid than previously assumed, with morphological plasticity potentially influencing taxonomic interpretations.

Another feature worthy of further research is the life strategy in Pseudanabaenales. Data from strains and sequences available in GenBank strongly suggest that Pseudanabaenales are predominantly confined to aquatic environments. However, we have successfully isolated a terrestrial subaerophytic strain from this group that grew epilithically at a cave entrance, indicating potential adaptability beyond true aquatic habitats. The strain occupied a rock that was wet even during the high summer season and dry weather, with constant moisture provided by condensed vapor coming out from the humid cave. Therefore, it is possible that future work will identify in aquatic habitats in the area. That said, the strain of *Portococcus sanctae‐mariae* produced a wide extracellular polysaccharide layer (Figure 1i) reminiscent of mucilaginous sheaths that retain water and protect the cells against desiccation in typical rock‐dwelling terrestrial cyanobacteria (Hauer et al., 2015). The species also exhibited radial thylakoid arrangement, which is not typical for Pseudanabaenales (Mareš et al., 2019). We speculate that it might be connected to the low light regime at the cave entrance (a snapshot measurement of irradiance at only 0.06 μmol photons · m^−2^ · s^−1^ was acquired upon sampling on a cloudy summer day around the noon), but further research is needed to understand this possible relationship. One could note that *P*. *sanctae‐mariae* is the semi‐terrestrial cyanobacterium appearing in the phylogenetic tree, aside from the ancestral *Gloeobacter* (Mareš et al., 2013). More likely, a yet unknown diversity of Pseudanabaenales is awaiting discovery in the still understudied terrestrial habitats.

Our study compared the results of a standard polyphasic taxonomic analysis based on rRNA gene sequencing (complemented with morphological and ecological characterization) to the results of a genome‐based analysis. We conclude that both types of analyses agreed in the definition of the species and genera using monophyletic clustering in phylogenetic trees (Johansen & Casamatta, 2005). They were further congruent in the results based on sequence identity parameters such as the pairwise p‐distance in the 16S rRNA gene and the ITS rRNA region (Tables 2, 3), compared to the genome‐derived ANI, AAI, and dDDH values (Table 4).

The current study has presented comparative structures of the entire ITS rRNA region (Figures S1–S5). This analysis showed major differences in ITS rRNA region structure between the five genera of Pseudanabaenales, indicating that the complete ITS rRNA region may be more broadly useful for distinguishing genera in the future. The variable presence of the V2 helix (absent in *Portococcus* and *Thalassoporum*, Figures S1, S4), the presence of a pre‐tRNA helix in *Tumidithrix elongata* and all species of *Portococcus* (S1, S5), and the absence of a V3 and D5 helix in *Pseudanabaena* and *Thalassoporum* (S3, S4) all indicated that it may be time to start examining the whole ITS rRNA region among genera to see how consistent the presence or absence of conserved helices is within and among genera. Presently, much of the modern taxonomy of cyanobacteria is based on molecular thresholds (16S gene similarity, ITS rRNA region dissimilarity, and ANI values). These have been effectively applied although they have often lacked clear discontinuities for reliable diagnoses between genera. The pre‐tRNA helices were a clear and useful synapomorphy separating *Portococcus* and *Tumidithrix* from all other cyanobacteria sequenced thus far. Likewise, the D5 helix in both *Pseudanabaenococcus* and *Portococcus* separated these two genera from almost all other genera (Figures S1, S2). The presence of unusually long and structurally complex D1‐D1′ and D5 helices in *Tumidithrix*, together with additional helices in that genus (Figure S5), served to separate this genus from all other cyanobacterial genera sequenced to date. Finding distinctive helices could provide autapomorphic or synapomorphic characters that more clearly diagnose genera and species. In the future, we hope others will explore the information in the whole ITS rRNA region as it could likely be a source of taxonomically diacritical information.

Early in the study of molecular systematics, the 5S rRNA gene was sequenced and reported for a number of taxa (Delihas et al., 1984; Hori & Osawa, 1987; Van den Eynde et al., 1990; Van den Eynde et al., 1988). More recent interest in this sequence resulted in comparisons of phylogenies constructed from 5S and 16S rRNA gene sequences (Moten et al., 2017). The 5S rRNA gene sequence was determined to separate genera, but phylogenies based on this gene were not in full agreement with the 16S rRNA gene phylogenies. We observed that the change in the basal clamp sequence of the 5S rRNA molecule in *Pseudanabaenococcus* and *Pseudanabaena* made it difficult to even identify the 5S rRNA gene sequence, and we thought it might be missing (Figure S6). Our report may help those less familiar with this region to identify it in future studies. We anticipate that this region may have taxonomic value as it is studied in greater detail with cyanobacterial species‐validated sequences. Differences even among the four species of *Portococcus* indicated that there is notable sequence diversity in this short region.

Additional autapomorphies such as the differences in the leader region and the 23S–5S ITS rRNA region sequence and helix structure, as well as clearly different source habitats of the isolated strains, were supportive for the new species and genus descriptions. That said, the genomic phylogeny was an invaluable addition for the understanding of evolutionary relationships above the genus level. This phylogeny allowed us to determine that the two new unicellular genera formed a monophyletic lineage (Figure 4) and that their unicellularity was likely acquired by a secondary evolutionary event, which would not have been resolved by the 16S rRNA gene tree alone (Figure 3).

One of our taxa, *Pseudanabaenococcus habilitatus*, was represented by two strains, SAG 2387 (the reference strain from which the holotype was prepared) and PCC 7502. Walter et al. (2017) previously proposed a new genus and species name for PCC 7502, *Leptovivax bogii*, but it was invalid under the ICN for the following reasons: (1) there was no illustration of the taxon (Article 44.2), (2) the descriptions were contained in a supplemental Word (docx) file in supplementary materials rather than in portable document format (PDF; Article 29.1), (3) no physical holotype specimen in a metabolically inactive state was prepared or deposited in a herbarium (Article 8.4), and (4) the description was demonstrably inadequate for future determination (Article 38B.2). Furthermore, the species was purportedly a new combination from *Synechococcus bogii*, but no citation to the basionym was given (Article 41.1), and it was based on an invalidly published name (Article 41.5). *Leptovivax bogii* was also invalid under the ICNP for the following reasons: (1) the name was not published in the *International Journal of Systematic and Evolutionary Microbiology* (Rule 27.1 and Rule 27 Note 1), (2) the species was a new combination, and the full reference to the authors and dates of valid publication for the basionym were not given (Rule 27.2b), (3) the type strain was not deposited in two publicly accessible culture collections in different countries (Rule 30.3b), (4) the description was demonstrably ambiguous and could not be critically identified for purposes of the precise application of the name of a taxon (Rule 31a; the description consists of the statement: “This species is characterized by β‐carboxysome” [data sheet 1] and the size of the genome was given with the number of coding regions), and (5) the name must be in Latin or Latinized words treated as Latin, and words from other languages should be avoided if Latin equivalents exist; *bogii* is based on the English word bog, and Latin has at least three words for bog, *lama*, *lustrum*, and *palus*, and an adjectival form exists: *paludosus* (Principle 3, Rule 6).

Walter et al. (2017) described 28 new genera and 32 new species names based on using whole‐genome sequences. All names proposed in their publication were invalid and have no standing in nomenclature. However, the name has subsequently appeared in other publications (Salazar et al., 2020; Walter et al., 2020). Related to this theme, there were two proposals to the International Committee on Systematics of Prokaryotes (ICSP) to use whole‐genome sequences as types for new prokaryotic taxa in place of axenic reference strains deposited in two collections in different countries (Whitman, 2016; Whitman et al., 2019). The ICSP voted to reject both proposals (Sutcliffe et al., 2020). Subsequently, researchers supporting these proposals prepared their own nomenclatural code, the *International Code of Nomenclature of Prokaryotes Described from Sequence Data* (SeqCode, see Whitman et al., 2022). Similarly, the proposal to use DNA sequences in place of types for microalgae (including cyanobacteria) was discussed by the botanical nomenclature committees(Turland, 2025) but was not supported in the latest version of ICN (Turland et al., 2025). We currently cannot recommend using SeqCode for publication of new names of cyanobacteria, especially those that have cultured strains. The only requirements under this code are that researchers have a name, a genomic sequence, and that the name is published in a book or journal. We consider these requirements to be inadequate for use in cyanobacterial taxonomy, since without descriptions, illustrations, strains, and ribosomal sequences, the names created under this code cannot be linked to nomenclatural types previously described under the ICN or ICNP.

In conclusion, the study of Pseudanabaenales has provided valuable insights into the evolutionary pathways and ecological adaptations of deep‐branching cyanobacteria. The identification of unicellular strains in this basal filamentous group and the discovery of their capacity to thrive in nonaquatic environments challenge previous assumptions about the ecological and morphological constraints of Pseudanabaenales. This ongoing exploration has not only enhanced our understanding of cyanobacterial diversity but also underscored the importance of integrative approaches in taxonomy, bridging gaps between molecular phylogenetics, genomics, morphology, and ecological context. This study has confirmed that whole‐genome sequencing could contribute not only to the modern polyphasic approach in cyanobacterial taxonomy, especially for the construction of phylogenetic trees more reliable at higher taxonomic levels, but also to a way to overcome the complex evolution of rRNA operons recently reported in several cyanobacterial taxa (Johansen et al., 2017; Villanueva et al., 2024). An analysis based on both classical polyphasic approach and whole‐genome sequencing of reference strains of cyanobacterial type species seems to be the most powerful and up‐to‐date tool available to dissect taxonomic relationships in cyanobacteria.

## AUTHOR CONTRIBUTIONS

**Otakar Strunecký:** Conceptualization (equal); data curation (equal); formal analysis (equal); investigation (equal); methodology (equal); resources (supporting); validation (supporting); visualization (equal); writing – original draft (equal). **Eliška Kozlíková‐Zapomělová:** Investigation (equal); resources (equal). **Jitka Jezberová:** Investigation (supporting); resources (supporting). **João Morais:** Data curation (supporting); investigation (supporting); resources (equal); writing – review and editing (supporting). **María Alicia Toledo Lemus:** Data curation (supporting); formal analysis (supporting); investigation (supporting); writing – original draft (supporting). **Lenka Štenclová:** Data curation (equal); investigation (equal); writing – review and editing (equal). **Jeffrey R. Johansen:** Data curation (equal); formal analysis (equal); investigation (supporting); writing – original draft (supporting); writing – review and editing (supporting). **Kateřina Čapková:** Resources (supporting); writing – review and editing (supporting). **Vitor M. O. Vasconcelos:** Funding acquisition (supporting); resources (supporting); supervision (supporting); writing – review and editing (supporting). **Jan Mareš:** Conceptualization (equal); data curation (equal); formal analysis (equal); funding acquisition (lead); investigation (equal); methodology (equal); project administration (equal); resources (equal); supervision (equal); validation (equal); visualization (lead); writing – original draft (equal).

## Supporting information


**Figure S1.** Whole ribosomal operon for *Portococcus lusitanicus* LEGE16609, with position of rRNA genes indicted in triangles. Note the existence of a pre‐tRNA helix, absence of a V2 helix, and presence of both a V3 helix, and an elongated D5 region.

**Figure S2.** Whole ribosomal operon for *Pseudanabaenococcus habilitatus* PCC 7502, with position of rRNA genes indicted in triangles. Note the absence of the pre‐tRNA helix, the presence of a V2 helix, a V3 helix, and an unbranched elongated D5 region.

**Figure S3.** Whole ribosomal operon for *Pseudanabaena catenata* USMAC16, with position of rRNA genes indicted in triangles. Note the absence of the pre‐tRNA helix, the presence of a V2 helix, and an unbranched elongated D4 region lacking V3 and D5 regions.

**Figure S4.** Whole ribosomal operon for *Thalassoporum mexicanum*, with position of rRNA genes indicted in triangles. Note the absence of a Pre‐tRNA helix and V2 helix, and an unbranched elongated D4 region lacking V3 and D5 regions.

**Figure S5.** Whole ribosomal operon for *Tumidithrix elongate* BACA0141, with position of rRNA genes indicted in triangles. Note the presence of a long pre‐RNA helix, a V2 helix, two pre‐Box‐B helices, and both a V3 and a large D5 helix. This ITS regions was notably larger than all others (829 nt).

**Figure S6.** Sequence and secondary structure of the 5S rRNA molecule for the five taxa described in this manuscript plus the comparator taxon *Pseudanabaena catenata*. Note the differing sequence for the start of the 5′ end, where *Portococcus* is 5′‐UCCU‐3′, but *Pseudanabaenococcus* and *Pseudanabaena* start with 5′‐GCUU‐3′.

**Figure S7.** Comparison of genome sizes among available genome assemblies in genera of Pseudanabaenales. Box plots indicate mean ± *SD* and extremes, points indicate outliers. The genome size *Pseudanabaenococcus* was significantly smaller from the other taxa (pairwise *t*‐tests, *p* < 0.01).

**Figure S8.** Comparison of genomic GC content among available genome assemblies in genera of Pseudanabaenales. Box plots indicate mean ± *SD* and extremes, points indicate outliers. The GC content in *Pseudanabaenococcus* was significantly lower from the other taxa (pairwise *t*‐tests, *p* < 0.01).

**Figure S9.** Uncollapsed 16S rRNA phylogenetic tree (Bayesian inference) of cyanobacteria, showing the clustering of genera within the order Pseudanabaenales, including two new genera, *Portococcus* and *Pseudanabaenococcus*, resolved as monophyletic sub‐clades. Branch supports are shown at the nodes.

**Figure S10.** Uncollapsed phylogenomic tree (Maximum Likelihood, 120 conserved bacterial proteins) of cyanobacteria, showing the clustering of families and genera within the order Pseudanabaenales. The two newly described genera, *Portococcus* and *Pseudanabaenococcus* are grouped in a monophyletic unicellular sub‐lineage within the family Pseudanabaenaceae. Branch supports are shown at the nodes.
