# Supplementary figures and images for: To multicellularity and back again: Description of two new coccoid genera (Portococcus gen. nov. and Pseudanabaenococcus gen. nov.) in the basal “filamentous” order Pseudanabaenales, Cyanobacteria
Source: J Phycol. 2026 Feb 17;62(1):234–55. doi: 10.1111/jpy.70130 (PMC12961181; doi:10.1111/jpy.70130)

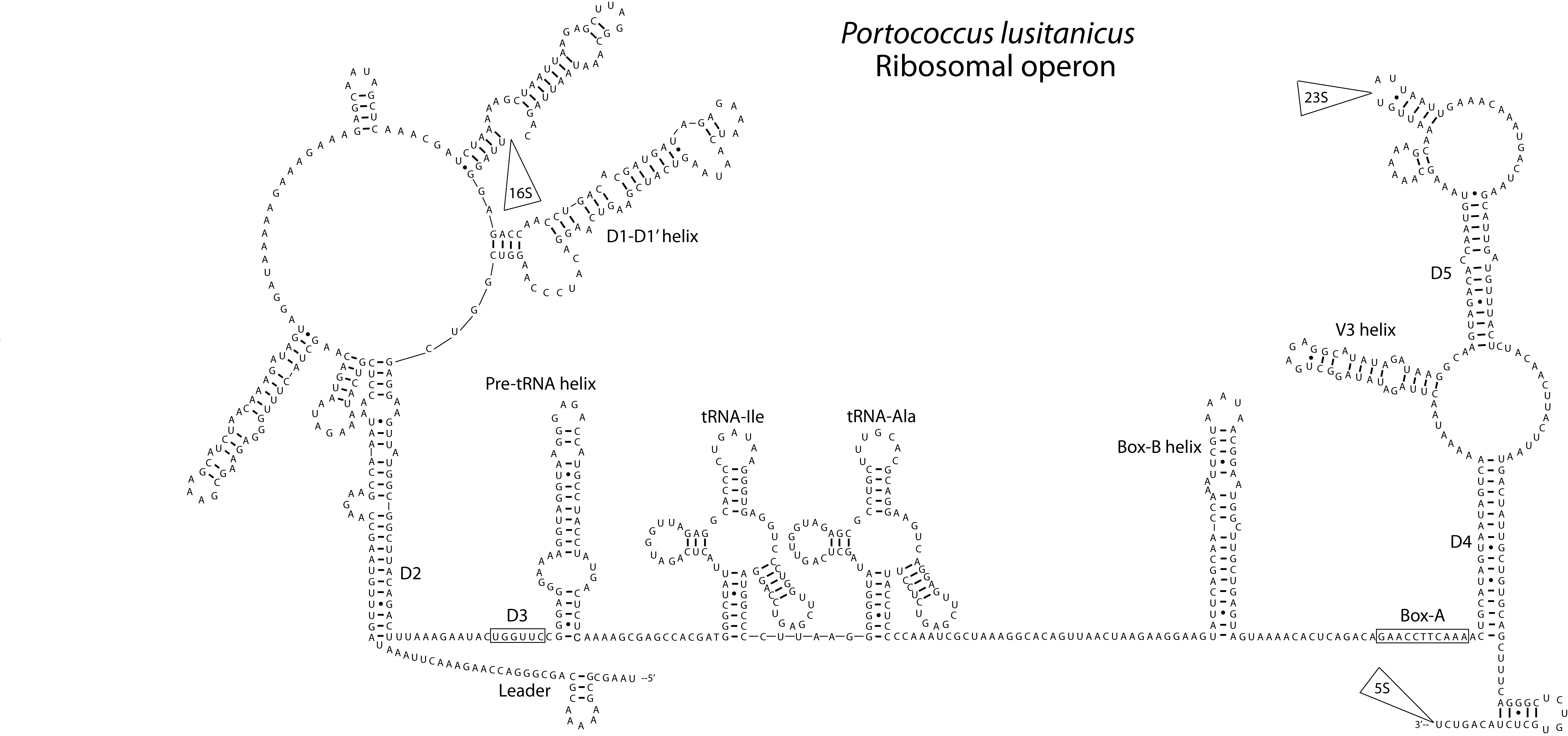

Supplement: Supplementary file 1 — Figure S1. Whole ribosomal operon for Portococcus lusitanicus LEGE16609, with position of rRNA genes indicted in triangles. Note the existence of a pre‐tRNA helix, absence of a V2 helix, and presence of both a V3 helix, and an elongated D5 region. [file JPY-62-234-s006.tif]

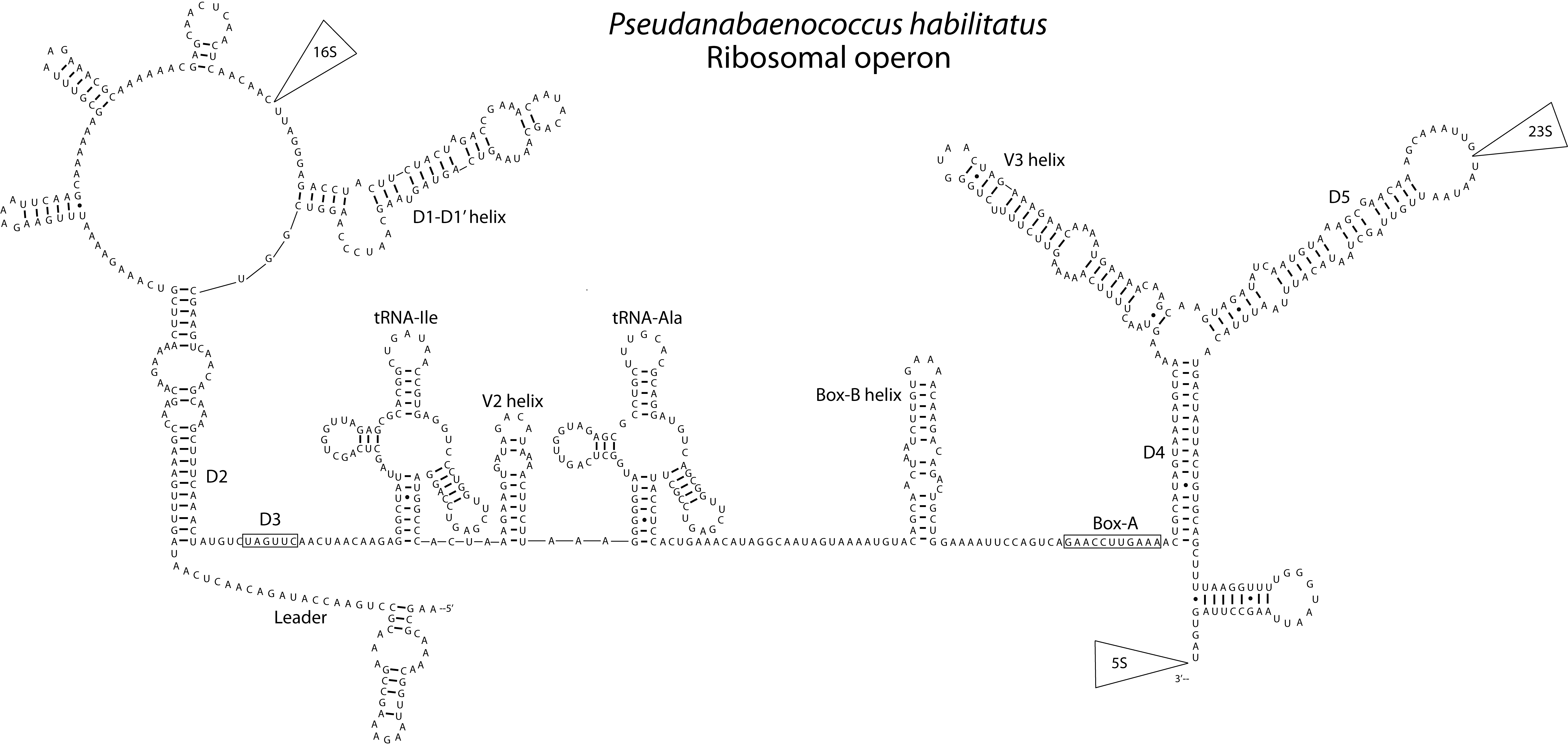

Supplement: Supplementary file 2 — Figure S2. Whole ribosomal operon for Pseudanabaenococcus habilitatus PCC 7502, with position of rRNA genes indicted in triangles. Note the absence of the pre‐tRNA helix, the presence of a V2 helix, a V3 helix, and an unbranched elongated D5 region. [file JPY-62-234-s010.tif]

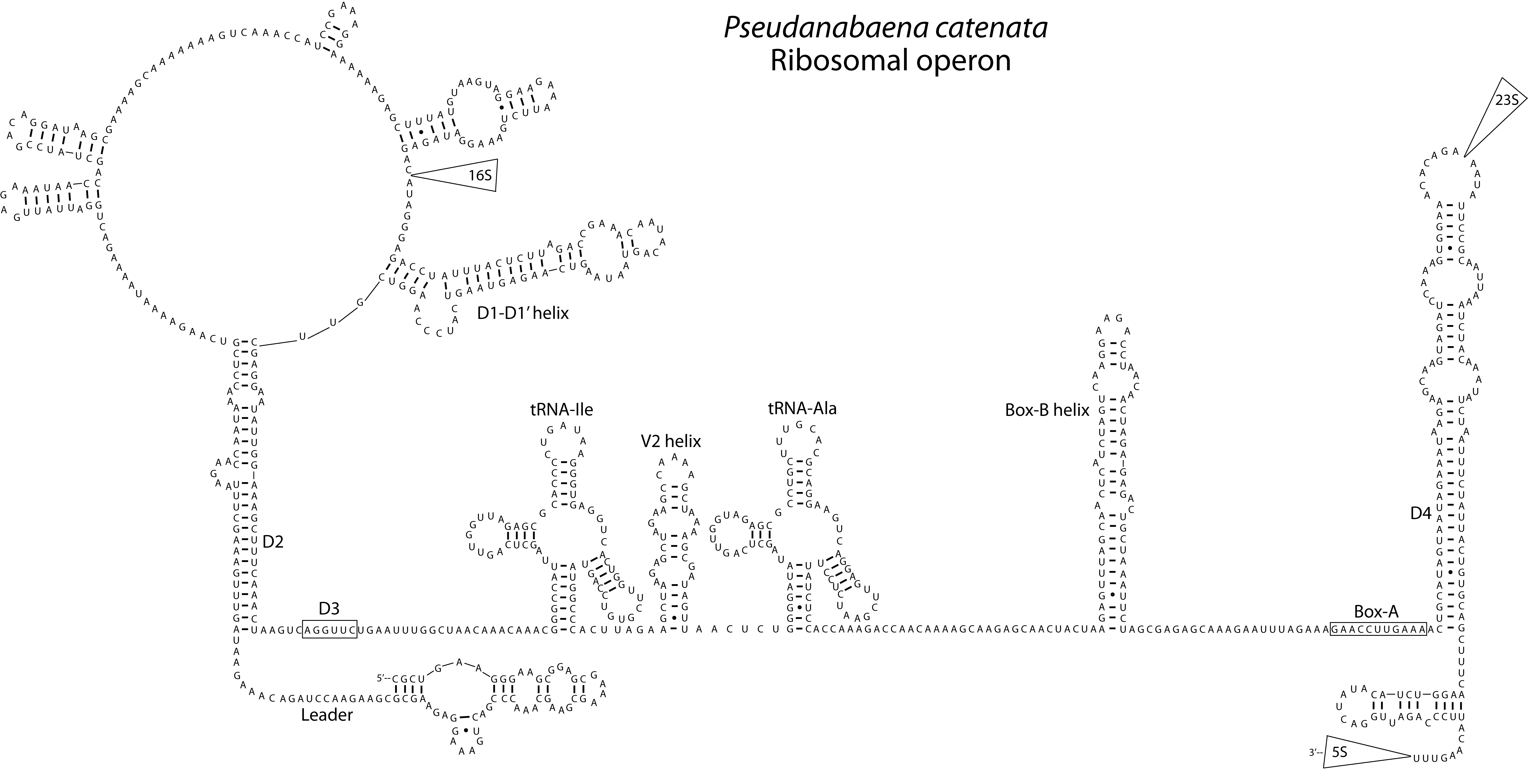

Supplement: Supplementary file 3 — Figure S3. Whole ribosomal operon for Pseudanabaena catenata USMAC16, with position of rRNA genes indicted in triangles. Note the absence of the pre‐tRNA helix, the presence of a V2 helix, and an unbranched elongated D4 region lacking V3 and D5 regions. [file JPY-62-234-s001.tif]

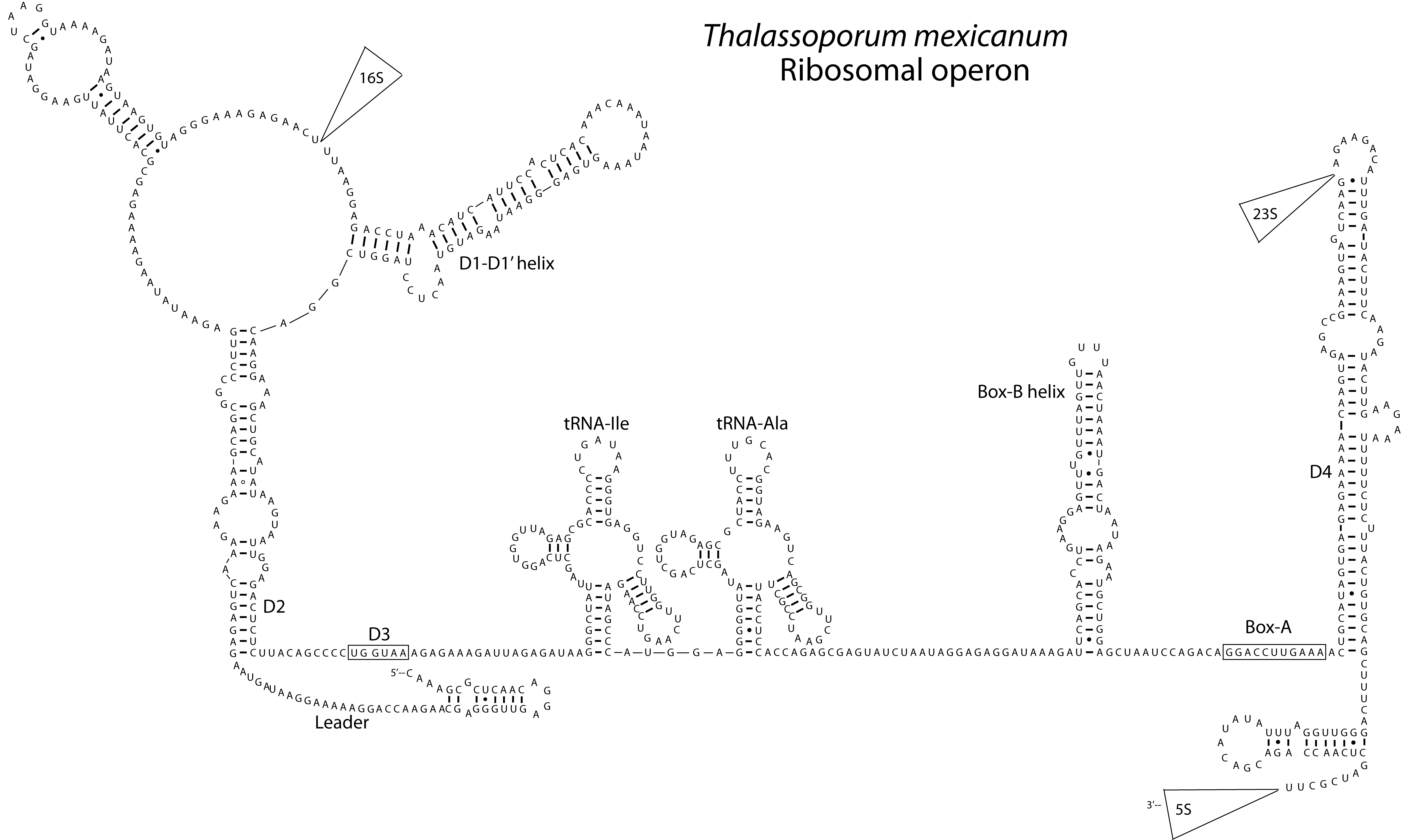

Supplement: Supplementary file 4 — Figure S4. Whole ribosomal operon for Thalassoporum mexicanum, with position of rRNA genes indicted in triangles. Note the absence of a Pre‐tRNA helix and V2 helix, and an unbranched elongated D4 region lacking V3 and D5 regions. [file JPY-62-234-s005.tif]

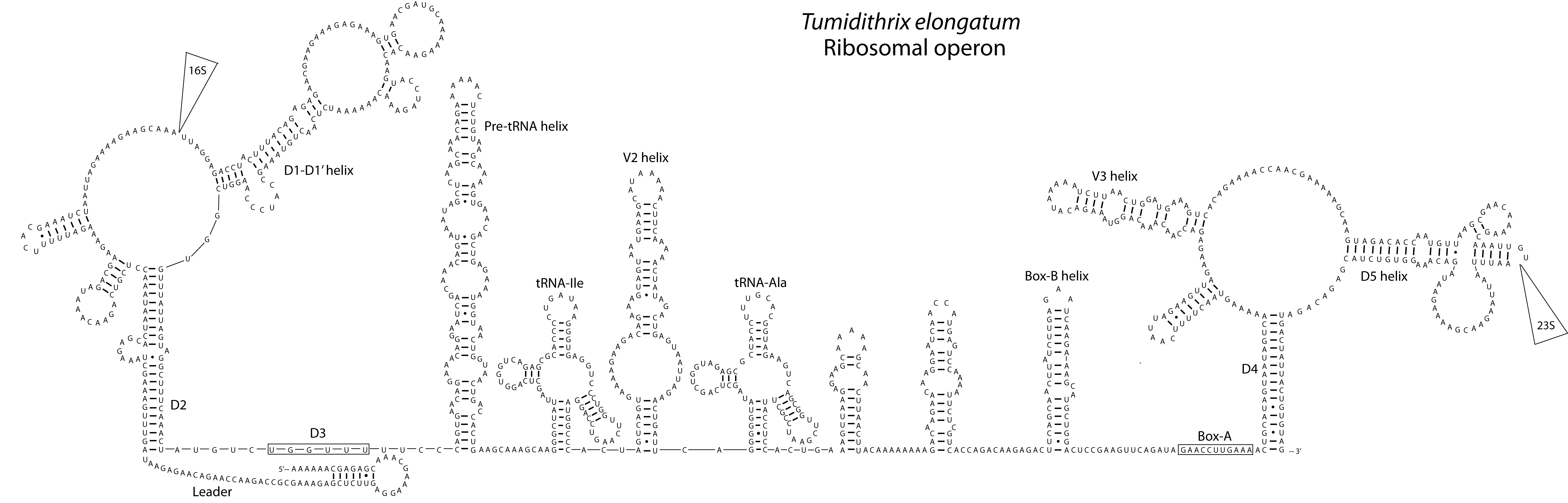

Supplement: Supplementary file 5 — Figure S5. Whole ribosomal operon for Tumidithrix elongate BACA0141, with position of rRNA genes indicted in triangles. Note the presence of a long pre‐RNA helix, a V2 helix, two pre‐Box‐B helices, and both a V3 and a large D5 helix. This ITS regions was notably larger than all others (829 nt). [file JPY-62-234-s008.tif]

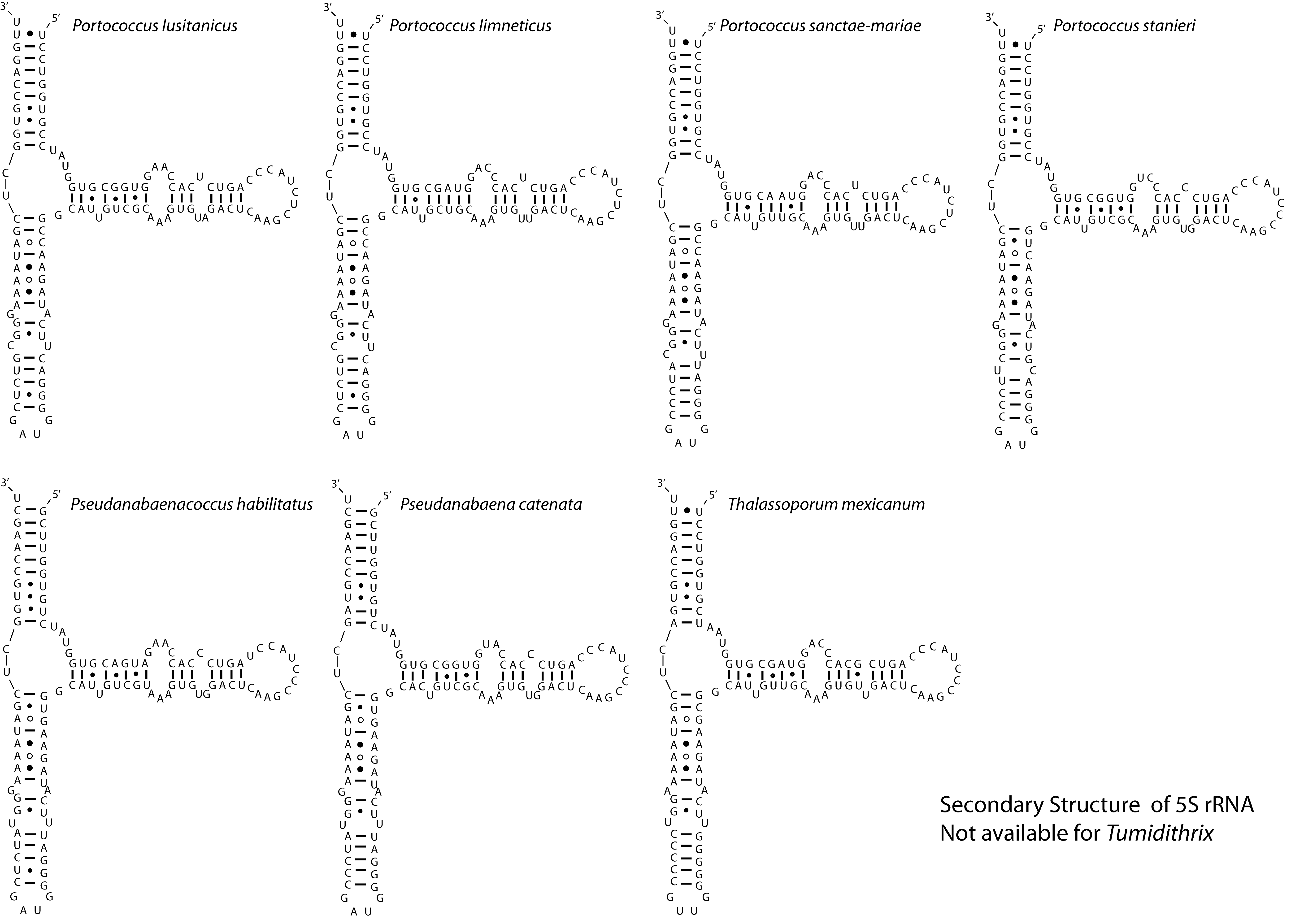

Supplement: Supplementary file 6 — Figure S6. Sequence and secondary structure of the 5S rRNA molecule for the five taxa described in this manuscript plus the comparator taxon Pseudanabaena catenata. Note the differing sequence for the start of the 5′ end, where Portococcus is 5′‐UCCU‐3′, but Pseudanabaenococcus and Pseudanabaena start with 5′‐GCUU‐3′. [file JPY-62-234-s007.tif]

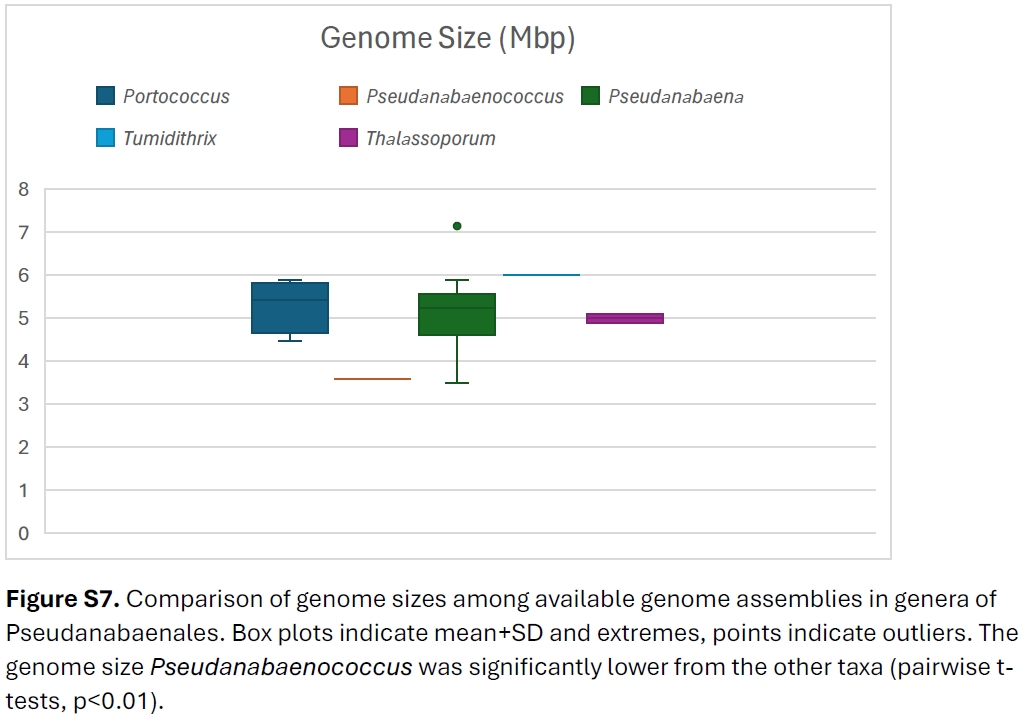

Supplement: Supplementary file 7 — Figure S7. Comparison of genome sizes among available genome assemblies in genera of Pseudanabaenales. Box plots indicate mean ± SD and extremes, points indicate outliers. The genome size Pseudanabaenococcus was significantly smaller from the other taxa (pairwise t‐tests, p < 0.01). [file JPY-62-234-s002.jpg]

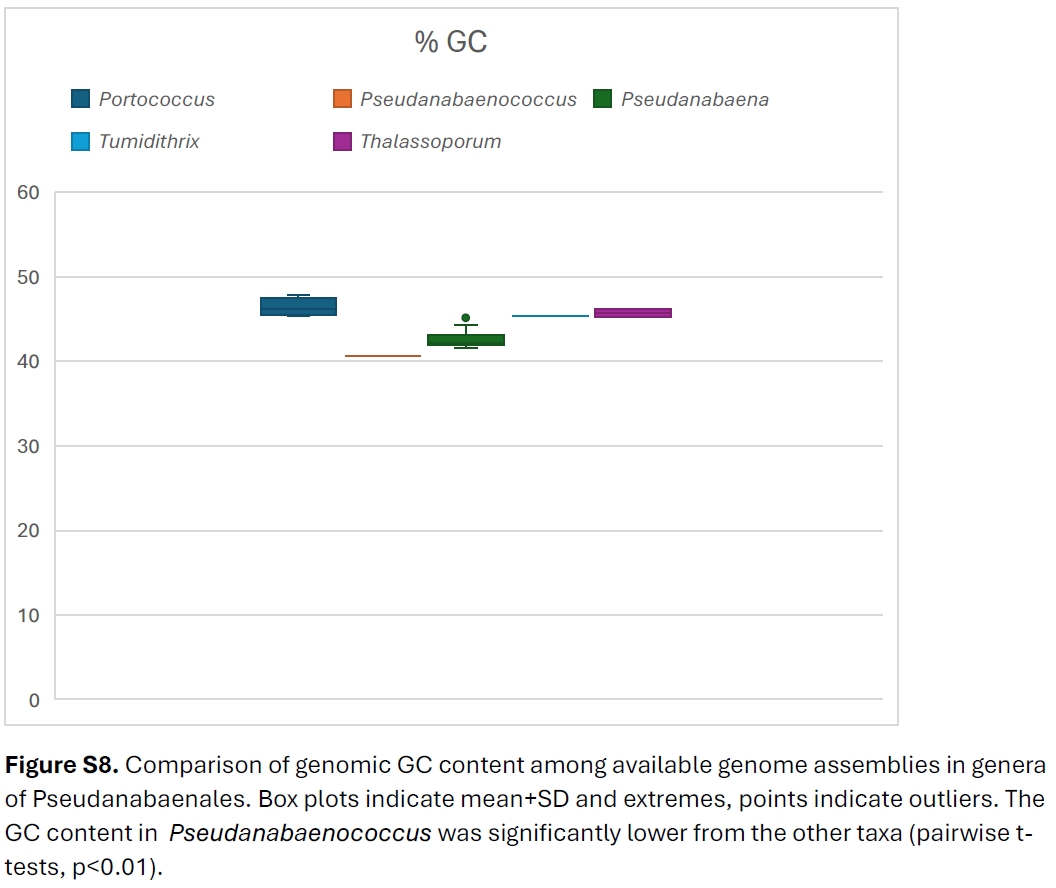

Supplement: Supplementary file 8 — Figure S8. Comparison of genomic GC content among available genome assemblies in genera of Pseudanabaenales. Box plots indicate mean ± SD and extremes, points indicate outliers. The GC content in Pseudanabaenococcus was significantly lower from the other taxa (pairwise t‐tests, p < 0.01). [file JPY-62-234-s004.jpg]

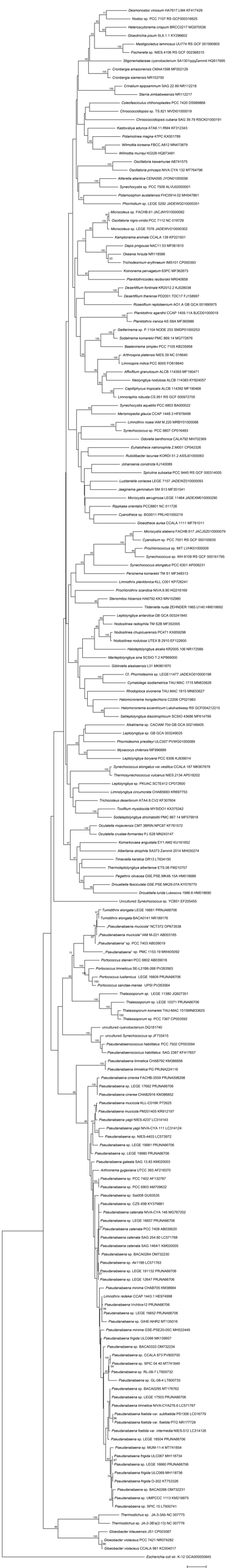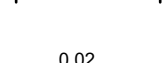

Supplement: Supplementary file 9 — Figure S9. Uncollapsed 16S rRNA phylogenetic tree (Bayesian inference) of cyanobacteria, showing the clustering of genera within the order Pseudanabaenales, including two new genera, Portococcus and Pseudanabaenococcus, resolved as monophyletic sub‐clades. Branch supports are shown at the nodes. [file JPY-62-234-s003.pdf]
