# Supplementary material for: To multicellularity and back again: Description of two new coccoid genera (Portococcus gen. nov. and Pseudanabaenococcus gen. nov.) in the basal “filamentous” order Pseudanabaenales, Cyanobacteria
Source: J Phycol. 2026 Feb 17;62(1):234–55. doi: 10.1111/jpy.70130 (PMC12961181; doi:10.1111/jpy.70130)

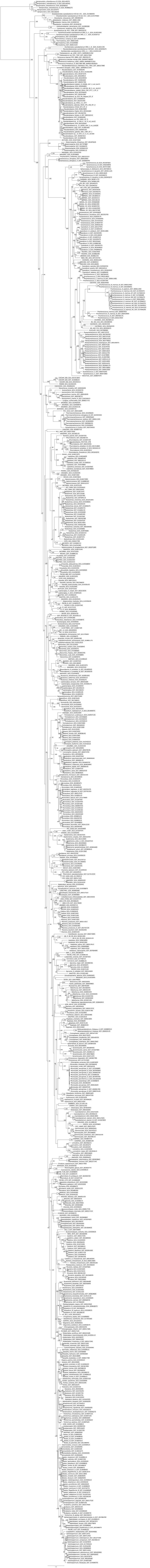

Supplement: Supplementary file 10 — Figure S10. Uncollapsed phylogenomic tree (Maximum Likelihood, 120 conserved bacterial proteins) of cyanobacteria, showing the clustering of families and genera within the order Pseudanabaenales. The two newly described genera, Portococcus and Pseudanabaenococcus are grouped in a monophyletic unicellular sub‐lineage within the family Pseudanabaenaceae. Branch supports are shown at the nodes. [file JPY-62-234-s009.pdf]
